# Different Infectivity of HIV-1 Strains Is Linked to Number of Envelope Trimers Required for Entry

**DOI:** 10.1371/journal.ppat.1004595

**Published:** 2015-01-08

**Authors:** Oliver F. Brandenberg, Carsten Magnus, Peter Rusert, Roland R. Regoes, Alexandra Trkola

**Affiliations:** 1 Institute of Medical Virology, University of Zürich, Zürich, Switzerland; 2 Institute of Integrative Biology, ETH Zürich, Zürich, Switzerland; Fred Hutchinson Cancer Research Center, United States of America

## Abstract

HIV-1 enters target cells by virtue of envelope glycoprotein trimers that are incorporated at low density in the viral membrane. How many trimers are required to interact with target cell receptors to mediate virus entry, the HIV entry stoichiometry, still awaits clarification. Here, we provide estimates of the HIV entry stoichiometry utilizing a combined approach of experimental analyses and mathematical modeling. We demonstrate that divergent HIV strains differ in their stoichiometry of entry and require between 1 to 7 trimers, with most strains depending on 2 to 3 trimers to complete infection. Envelope modifications that perturb trimer structure lead to an increase in the entry stoichiometry, as did naturally occurring antibody or entry inhibitor escape mutations. Highlighting the physiological relevance of our findings, a high entry stoichiometry correlated with low virus infectivity and slow virus entry kinetics. The entry stoichiometry therefore directly influences HIV transmission, as trimer number requirements will dictate the infectivity of virus populations and efficacy of neutralizing antibodies. Thereby our results render consideration of stoichiometric concepts relevant for developing antibody-based vaccines and therapeutics against HIV.

## Author Summary

Our estimates of the HIV-1 entry stoichiometry, that is the number of envelope glycoprotein trimers needed to mediate fusion of viral and target cell membrane, close an important gap in our understanding of the HIV entry process. As we show, stoichiometric requirements for envelope trimers differ between HIV strains and steer virus entry efficacy and virus entry kinetics. Thus, the entry stoichiometry has important implications for HIV transmission, as demands on trimer numbers will dictate the infectivity of virus populations, target cell preferences and virus inactivation by trimer-targeting inhibitors and neutralizing antibodies. Beyond this, our data contribute to the general understanding of mechanisms and energetic requirements of protein-mediated membrane fusion, as HIV entry proved to follow similar stoichiometries as described for Influenza virus HA and SNARE protein mediated membrane fusion. In summary, our findings provide a relevant contribution towards a refined understanding of HIV-1 entry and pathogenesis with particular importance for ongoing efforts to generate neutralizing antibody based therapeutics and vaccines targeting the HIV-1 envelope trimer.

## Introduction

To infect cells, HIV-1 virions need to fuse their membrane with the target cell membrane, a process triggered by the viral envelope (env) glycoprotein trimer [1], [2]. Due to its key function in the virus life cycle and as prime target for neutralizing antibodies and entry inhibitors, analyses of env trimer structure and function remain in the focus of current HIV vaccine and drug research [3]–[5]. Each env trimer consists of three heterodimeric protomers, composed of the non-covalently associated gp120 surface and gp41 transmembrane subunits. Binding of gp120 to the primary receptor CD4 on target cells triggers conformational changes in gp120 that expose the binding site of a co-receptor, most commonly CCR5 or CXCR4 [6]. Subsequent co-receptor binding activates the gp41 transmembrane subunits, which triggers a prototypic class I fusion process via insertion of the N-terminal fusion peptides into the target cell membrane. Refolding of the gp41 N- and C-terminal heptad repeat regions into six-helix bundles drives approximation and fusion of viral and target cell membranes [1], [7], [8].

While the HIV entry process has been defined in considerable detail, we currently lack information on the stoichiometric relations of interacting molecules. Likewise, the thermodynamic requirements of membrane fusion pore formation and pore enlargement, enabling passage of the viral core into the target cell cytoplasm, are only partially understood [9]–[11]. The energy required for the entry process is generated by structural rearrangements of the envelope trimer that follow receptor binding [7], [8], [12]. How many trimers must engage in receptor interactions (a number referred to as stoichiometry of entry) [13]–[15] in order to elicit the required energy to complete fusion has not been conclusively resolved. Whether HIV needs one or more trimers to complete entry will strongly influence virion infectivity and efficacy of neutralizing antibodies targeting the trimer. Previous studies resulted in contradicting stoichiometry estimates, suggesting that either a single trimer is sufficient for entry [13] or that between 5 to 8 trimers are required [14], [15]. In comparison, for Influenza A virus, which achieves membrane fusion through the class I fusion protein hemagglutinin (HA), postulated necessary HA trimer numbers range from 3 to 4 [16]–[18] to 8 to 9 [13]. Calculations based on the energy required for membrane fusion suggested that indeed the refolding of a single HIV envelope trimer could be sufficient to drive entry [7], [8]. Numerous lines of evidence however suggest that several env-receptor pairings are commonly involved in the HIV entry process. Electron microscopy analysis of HIV entry revealed the formation of an “entry claw” consisting of several putative env-receptor pairs [19], which is supported by biochemical analyses indicating that the number of CCR5 co-receptors needed for virus entry differs among HIV-1 isolates and requires up to 6 co-receptors [20], [21].

Precise delineation of the stoichiometry of entry, as we present it here, substantially contributes to our understanding of HIV pathogenesis by defining a viral parameter that steers virus entry capacity, potentially shapes inter- and intra-host transmission by setting requirements for host cell receptor densities, and by defining stoichiometric requirements for virion neutralization. The latter is of particular importance considering the ongoing efforts to generate neutralizing antibody based therapeutics and vaccines targeting the HIV-1 entry process [3], [4], [22].

## Results

### The number of envelope trimers required for entry differs among HIV-1 isolates

To estimate the stoichiometry of entry (in the following referred to as T) we employed a previously described combination of experimental and modelling analyses [13]–[15]. Our strategy centers on the analysis of env pseudotyped virus stocks carrying mixed envelope trimers consisting of functional (wt) and dominant-negative mutant env, where a single dominant-negative env subunit incorporated into a trimer renders the trimer non-functional. We included envs of 11 HIV-1 strains in our analysis covering subtypes A, B and C and a range of env characteristics such as primary and lab-adapted strains, different co-receptor usage and different neutralization sensitivities (Table 1).

**Table 1:** HIV-1 strains and mutants.

Strain	Subtype	Trimer Numbers^a^	R508S/R511S expression level^b^	V513E expression level^b^
SF162	B	16.0	95	90
NL4-3	B	13.5	85	90
JR-FL	B	11.8	85	95
RHPA	B	13.8	90	95
REJO	B	10.5	100	100
AC10	B	15.0	100	100
P3N	B	20.3	100	100
ZA110	B	15.0	80	100
CAP88	C	7.3	100	100
ZM214	C	6.7	100	100
BG505	A	9.5	95	100

Eleven HIV-1 strains from subtypes A, B and C were employed in this study. Mean virion trimer numbers and expression levels of the dominant-negative R508S/R511S and V513E mutants of the utilized virus preparations are shown.

a Mean virion trimer numbers as estimated by gp120/p24 ELISA of purified HIV-1 pseudoparticle stocks. Data are means of 2 to 3 independent experiments.

b The expression level of the dominant-negative mutants is recorded as percentage of the corresponding wt env based on gp120 quantification by ELISA of purified HIV-1 pseudoparticle stocks.

To derive estimates of T from mixed trimer experiments two key parameters need to be considered: the mean virion trimer numbers and the distribution of virion trimer numbers across a virion population [14], [15]. To assess virion trimer numbers, we determined p24 and gp120 content of purified virus stocks by ELISA. Although only an approximation, as also partially shed and non-functional trimers are accounted for, this analysis yielded upper limits of virion trimer content. We observed between 6 to 20 trimers per virion among the 11 strains probed (Table 1), which is in close agreement with previous estimates of HIV-1 virion trimer content [23]–[28]. While trimer incorporation into pseudotyped particles may be lower than on replication-competent virus [29], this does not preclude estimation of T as trimer content is controlled for in our mathematical analysis. Since available experimental data do not provide information on the distribution of trimers across virion populations (i.e. frequencies of virions with a given trimer number in the population), we utilized previously determined trimer number variation across virions in our modeling [26].

Key in our experimental design are dominant-negative env mutants. To obtain robust estimates of T we performed the experiments with two individual env mutations that both lead to a complete loss in entry capacity, either by a mutation of the Furin cleavage site (R508S/R511S) [13], [30], [31] or a mutation of the gp41 fusion peptide (V513E) [13], [32]. Importantly, expression levels of the mutant envs were in the range of 80 to 100% of the corresponding wt envs (Table 1), ascertaining that during mixed trimer experiments env expression levels on virions follows the ratio of wt and mutant env plasmids transfected into virus producer cells.

**Figure 1:**
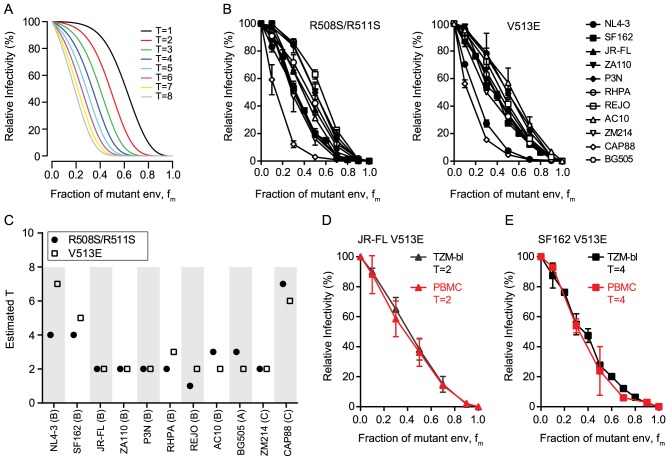
HIV-1 strains differ in the number of trimers required for entry. (A) Theoretical predictions of relative virus infectivity over the fraction of dominant-negative mutant env (f_m_) according to our model. Curves for T ranging from 1 to 8 are shown assuming the trimer number distribution across virions to follow a discretized Beta distribution with constant mean 12.95 and variance 45 [15]. (B) Relative infectivity of mixed trimer infection experiments with 11 HIV-1 strains using the R508S/R511S (left) and V513E (right) dominant-negative env mutants. Infectivity of pseudotyped virus stocks expressing the indicated ratios of wild type and dominant-negative mutant envs was measured on TZM-bl reporter cells. Infectivity of virus stocks containing solely wt envelope were set as 100%. Data depict mean and SD from 2 to 4 independent experiments. For each virus the individual curve fits resulting from the model to the obtained data were evaluated (S1 Fig.). (C) Mathematical estimates of T derived from the data shown in (B). The R508S/R511S (black circles) and V513E (open squares) mutations were analyzed individually. Bootstrap analyses demonstrating the robustness of the obtained estimates of T are shown in S1 Fig. (D and E) Mixed trimer virus stocks for strain JR-FL (D) and SF-162 (E) carrying the V513E mutation were assayed on healthy donor PBMC and compared to data obtained with TZM-bl target cells. Data depict mean and SD from 2 independent experiments.

To assess T, mixed trimer expressing pseudovirus stocks of each strain were generated by transfecting producer cells with different ratios of mutant and wt env plasmids ranging from 0 to 100% of the dominant-negative mutant env. The resulting virus stocks were probed for infectivity on TZM-bl reporter cells and infectivity was plotted as function of mutant env content (Fig. 1A and B). Based on our model [15], differences in T result in different infectivity plots in this graphical analysis (Fig. 1A). Intriguingly, the 11 envs showed variable patterns suggesting that the strains differ in T (Fig. 1B). The mixed trimer infectivity data (Fig. 1B) and the mean virion trimer numbers (Table 1) were then used to infer T of each strain by our model. This resulted in estimates of T ranging from 1 to 7 trimers for the 11 HIV strains tested (Fig. 1C, S1 Table and S1 Fig.), with the majority of strains requiring 2 to 3 trimers for entry. Of note, the data derived with the V513E and R508S/R511S mutant returned closely matching results with identical estimates for T (n = 4) or estimates differing only by 1 (n = 6) (Fig. 1C). The only higher discrepancy was observed for the highly neutralization sensitive, T cell line adapted strain NL4-3 where the two env mutations appeared to have individual effects on the readout but both yielded estimates of T that were amongst the highest in the env panel (T = 7 with V513E and T = 4 with R508S/R511S).

To verify that the mathematical approach (in the following referred to as “basic model”) provides a valid estimate of T, we probed several alternative analyses of the data shown in Fig. 1B. These analyses incorporate previously described extensions of the basic model that account for additional parameters that could potentially influence data acquisition and analysis [15]. The model extensions showed for the majority of strains a significantly improved curve fit to the experimental data (S1 Table). However, these analyses frequently yielded highly divergent values of T and the additional model parameters included in the model extensions for the two dominant-negative mutants of the same strain (S1 Table and exemplified for the CAP88 Env in S2 Fig.). This was in stark contrast to the basic model where the two independent T estimates of each strain were with few exceptions in close agreement (Fig. 1C and S1 Fig.). As we can safely assume that the parameter estimates for the two different mutations of the same strain should be similar we can reject the model extensions. A further indication that the model extensions we probed are not valid in the context of our analysis was that the derived estimates of T were in many cases implausibly high whereas the basic model generated estimates that fit the described range of trimer levels on HIV virions. We are thus confident that the basic model we utilize for the estimation of T is valid and provides robust estimates.

The mean virion trimer number of the probed virus stocks is an important model parameter in our analysis of T and fluctuations in the mean virion trimer number may therefore influence the estimates. To test the influence of mean virion trimer number variation on our estimates of T we performed additional data analyses where instead of the measured individual trimer numbers (Table 1), identical mean trimer numbers for all 11 strains were assumed. We chose 3 values for this comparison that covered the range of trimer numbers measured across our panel: mean trimer numbers of 5 and 26 (representing the lowest and highest trimer contents measured in individual experiments) and a mean trimer number of 13, the mean of trimer numbers measured across our virus panel. Applying these trimer numbers to our data set we obtained estimates of T ranging from 1 to 17 trimers (S3A Fig.).

To determine the mean virion trimer numbers of the virus stocks we measured gp120 and p24 contents. This allows to derive virion numbers based on previously reported estimates of 1200 to 2500 p24 molecules per virion [23], [24], [33]. We chose an average estimate of 2000 p24 molecules per virion to derive the mean trimer numbers shown in Table 1. To investigate the influence of p24 assumptions on our analysis we also tested a higher p24 content estimate of 2400 molecules per virion as recently reported [33], consequently yielding 20% higher mean virion trimer numbers across all viruses. Employing these 20% higher trimer numbers in our analysis had only a modest effect on the T estimates yielding identical or slightly higher (mostly by one trimer) estimates of T (S3B Fig.). While these analyses confirm that absolute values of T vary depending on the mean virion trimer number assumed for the analysis, the differences in T among the 11 strains persisted, highlighting that they reflect qualitative entry properties of the respective envs. Hence, independent of the absolute mean trimer numbers, differences in T between viral strains can be detected by our approach.

As a further assay verification we tested the influence of target cells on our estimation of T. We reasoned that if our experimental approach truly measures the stoichiometry of entry, then the obtained data should be a sole function of the envelope trimer and not be influenced by target cell type and receptor density. We thus chose TZM-bl cells as target cells for their known reproducible performance and good signal to noise ratio in the luciferase reporter readout. Since these engineered cells overexpress the entry receptors of HIV and thus do not reflect features of physiological relevant target cells, we sought to verify that the obtained T estimates are indeed independent of the target cells used. To this end we chose two envelopes which yielded a low T estimate (primary isolate JR-FL, T = 2) and a high T estimate (lab-adapted strain SF162, T = 4 to 5) on TZM-bl cells and repeated the estimation of T on PBMC as target cells (Fig. 1D and E and S4 Fig.). As anticipated, we obtained for both viruses almost identical curves and T estimates as with the TZM-bl reporter cells, confirming that estimates of T are truly independent of the target cell type. Hence, use of TZM-bl cells for our assay setup is appropriate and the estimated T values are valid for physiologically relevant target cells of HIV-1.

### Increased virus entry fitness is reflected by low entry stoichiometry

**Figure 2:**
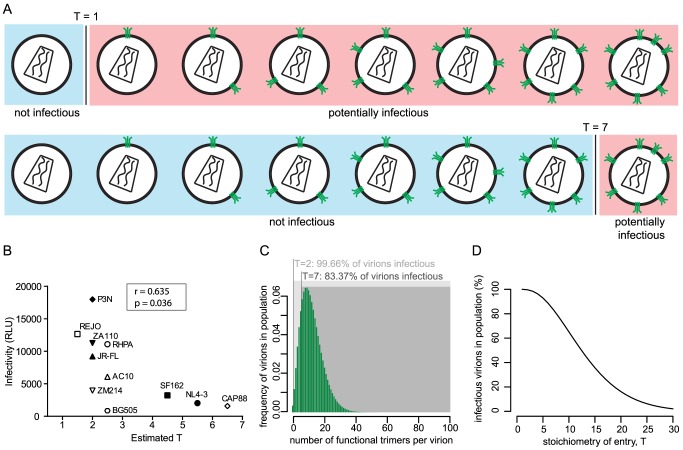
The entry stoichiometry governs virus population infectivity. (A) Scheme depicting the influence of the entry stoichiometry on virus population infectivity. Different Ts (exemplified here: T = 1 and T = 7) will determine the minimum number of trimers that a virion requires in order to be infectious. (B) Correlation analysis (Pearson) of virus strain infectivity (measured by infection of TZM-bl reporter cells and expressed in arbitrary relative light units (RLU) per µl of virus stock) and the estimated T (plotted as mean of the independent R508S/R511S and V513E estimates shown in Fig. 1C). Virus infectivities are depicted as mean values derived from 3 independent experiments. (C) Mathematical modeling to investigate the influence of entry stoichiometry on virion population infectivity. The data depict how T = 2 and T = 7 translate into different fractions of a virion population being potentially infectious, in dependence on the trimer number distribution across the virion population. As shown in (D), the overall infectivity of a virus population decreases with increasing T. For (C) and (D) we assumed the trimer number distribution across virions to follow a discretized Beta distribution with constant mean 12.95 and variance 45 [15].

The entry stoichiometry of a strain can be expected to influence virus population infectivity as strains with a low T will benefit from a higher proportion of the virus population carrying the required minimum trimer number (Fig. 2A). To directly probe the influence of T on virus infectivity we assessed the *in vitro* infectivity of the 11 HIV-1 strains in our panel. Of note, in the context of pseudoviruses infectivity is solely determined by the Env genes. Intriguingly, infectivity proved to be inversely correlated with T (r = −0.635, p = 0.036; Fig. 2B) indicating that strains that accomplish entry with low T are more infectious than strains with high T. Of note, we observed very divergent infectivities also for strains with very similar estimates of T (Fig. 2B). This is likely caused by different mean trimer numbers of the strains, as the mean trimer number in conjunction with T dictates virion population infectivity (Fig. 2A, C and D). For instance, amongst the viruses with T = 2 strain P3N has the highest infectivity and highest mean virion trimer number (20.3) whereas ZM214, the strain with lowest infectivity also has the lowest mean virion trimer number (6.7) measured across these viruses (Table 1). It can expected that additional factors beyond T and trimer numbers, such as propensity to shed gp120 or differential affinity for CD4, which are not covered by our analysis, may further contribute to different infectivity of the strains.

To investigate the interplay between entry stoichiometry and infectiousness of a virus population in more detail, we performed mathematical analyses of the relation between entry stoichiometry and trimer numbers per virion of a virus population. We found that indeed the entry stoichiometry steers virus population infectivity, with a higher entry stoichiometry resulting in a lower fraction of potentially infectious virions (Fig. 2C and D). Hence, the T of a strain and the therewith linked entry capacity may potentially contribute to the infectious to non-infectious particle ratio which is known to be low for HIV-1 [24].

### Perturbation of trimer integrity induces changes in entry stoichiometry

**Figure 3:**
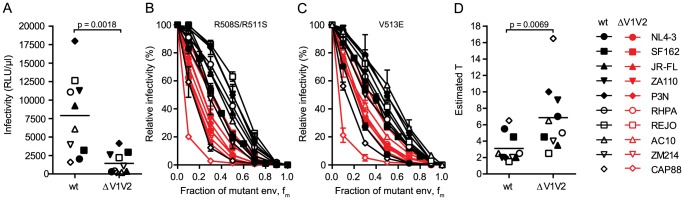
V1V2 deletion impairs virus infectivity and is reflected by a high stoichiometry of entry. (A) Comparison of infectivity of pseudoviruses expressing wt and V1V2-deleted envs upon infection of TZM-bl cells. Data points depict mean values of luciferase reporter activity per µl virus stock measured in 3 independent experiments. The p-value was calculated by a paired t-test. (B and C) Relative infectivity of mixed trimer infection experiments of 10 wt envs and their V1V2 deleted variants using the R508S/R511S (B) and the V513E (C) dominant-negative mutations are shown. Infectivity of pseudotyped virus stocks expressing the indicated ratios of dominant-negative mutant envs was measured on TZM-bl cells. Infectivity of virus stocks containing solely functional envelope were set as 100%. Data depict mean and SD from 2 to 4 independent experiments. (D) Estimates of T for the wt and V1V2-deleted envs derived from mixed trimer experiments. Data points are the mean of the individual estimates of T obtained with the R508S/R511S and V513E dominant-negative mutations. The p-value was calculated by a paired t-test.

To further explore the relation between virus infectivity and T we analyzed envs with deletions of the gp120 variable loops 1 and 2 (V1V2) and compared them to the matching wildtype envs. As we and others have previously shown, V1V2 deletion causes a dramatic reduction of virus infectivity through impairment of trimer integrity (Fig. 3A and [34]–[38]). When we probed T and compared the infectivity curves of the wt and V1V2-deleted env pairs, we observed distinct curve shifts of the V1V2-deleted envs across the majority of strains (Fig. 3B and C). Indeed, T of the V1V2-deleted envs proved significantly increased compared to the matching wt envs (Fig. 3D; mean T of 3.1 for wt envs versus mean T of 6.85 for V1V2-deleted envs; paired t test p = 0.0069). Importantly, this reduction in entry efficiency and the ensuing high estimates for T upon V1V2 deletion are not simply caused by reductions in env content of these virions, as V1V2-deleted env is expressed to similar levels on virions as the corresponding wt env (80–100% of wt, S5 Fig.). While expression levels of trimers certainly influence the estimates of T, we verified that the observed env content reduction of V1V2 deleted viruses was too low to inflict an overestimation of T (S5 Fig.) highlighting that indeed functional properties and not quantity of the respective wt and ΔV1V2 envelopes are decisive in defining T.

To further investigate the interplay between trimer numbers and T we produced pseudoviruses which expressed JR-FL wt and JR-FL ΔV1V2 with a deletion of the gp41 cytoplasmic tail (CT) as this is known to lead to an increased incorporation of trimers into virions [39], [40]. Indeed, CT deletion resulted in approximately 2-fold increased levels of trimers on virions (S6A–S6B Fig.). In support of the strong interplay between virion trimer numbers and infectivity thresholds defined by T, the infectivity of both viruses upon CT deletion was increased (S6C Fig.). Intriguingly, the increase in infectivity upon CT deletion was higher for JR-FL ΔV1V2 (9-fold) compared to JR-FL wt (2-fold), highlighting that envelopes with a reduced entry capacity, as here JR-FL ΔV1V2, benefit more if virions carry higher trimer numbers and thus meet the stoichiometric requirements for entry (S6D–S6E Fig.).

### Virus entry kinetics reflect requirements for trimer numbers during entry

**Figure 4:**
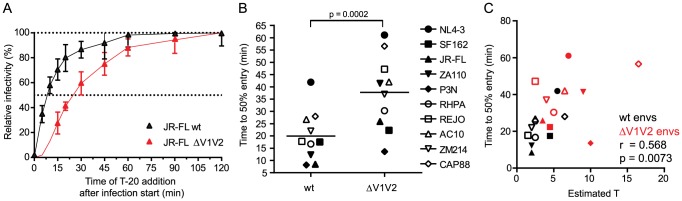
Virus entry kinetics correlate with the stoichiometry of entry. (A) Entry kinetic curves for JR-FL wt and JR-FL ΔV1V2. Synchronized pseudovirus infection of TZM-bl cells following spinoculation was terminated by addition of T-20 at the indicated timepoints. Infectivity reached after 120 minutes was set as 100% and all data were normalized relative to this value. Data are mean and SD from 3 independent experiments. (B) Half maximal entry time for wt and V1V2-deleted envs was calculated from kinetic profiles shown in Fig. 4A and S7B Fig. Time (in minutes) required to reach 50% entry into target cells is depicted. Data shown are means derived from 2 to 4 independent experiments. The p-value was calculated by a paired t-test. (C) Correlation analysis (Pearson) of wt (black symbols) and V1V2-deleted env (red symbols) half-maximal entry time and estimated T.

The number of trimers required for HIV entry likely influences virus infectivity in many ways. Besides determining a threshold trimer content that renders virions infectious, different T's could also manifest in different kinetics of the entry process as viruses with higher T may require more time to recruit and engage the necessary number of trimer-receptor pairings. To determine virus entry kinetics we employed a time-of-inhibitor addition experiment to derive the time required per virus strain to reach 50% of entry into target cells (Fig. 4A and S7A–S7B Fig.). Synchronized infection following spinoculation and temperature arrest in this assay setup allows assessment of entry kinetics solely as factor of envelope function post attachment to the target cells. When comparing the entry kinetics of the wt and V1V2-deleted strains we found that V1V2-deleted envs showed significantly delayed entry into target cells (Fig. 4B; mean times to 50% entry 19.9 minutes for wt envs and 37.7 minutes for V1V2-deleted envs; paired t-test p = 0.0002). As stated above, a potential explanation for this is that more time is required for V1V2-deleted strains to assemble a sufficient number of trimers in the virus-target cell contact zone to achieve entry. Indeed, we found a strong correlation between the estimated T and half-maximal entry time when all viruses, wt and V1V2-deleted strains, were analyzed (Fig. 4C; r = 0.568, p = 0.0073) but also for wt envs alone (r = 0.649, p = 0.0307), highlighting that entry stoichiometry and entry kinetics are tightly linked. The fact that we observe a significant correlation between estimated T and half-maximal entry time does however not exclude that additional processes beyond the recruitment of the necessary number of trimer-receptor pairings also influence the entry kinetics. Rates of CD4 and co-receptor binding and speed of the ensuing conformational rearrangements may differ between strains [41] and thereby contribute to the overall variance in entry kinetics. Additionally, for virions with a high T it must be considered that formation of the required number of contacts with the target cell may need longer time periods during which virions may detach again or decay before entry is completed [42].

### Naturally occurring loss of the N160 glycosylation site influences the stoichiometry of entry

**Figure 5:**
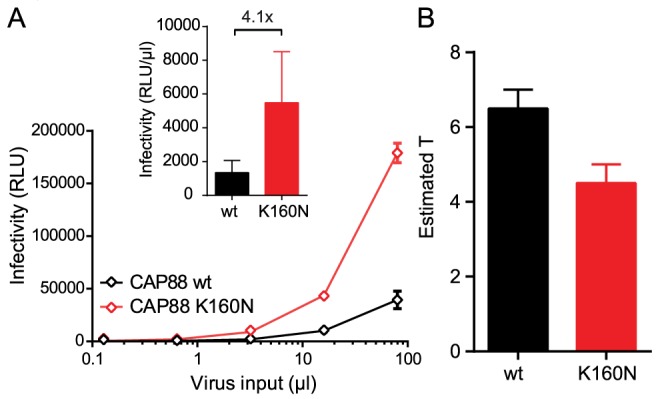
Loss of the N160 glycosylation site in gp120 steers the stoichiometry of entry and virus infectivity. (A) Titration of CAP88 wt (black) and CAP88 K160N (red) pseudoviruses on TZM-bl reporter cells. Data shown are mean and SD of luciferase reporter activity upon infection measured in 2 independent experiments. Inset: Infectivity comparison of CAP88 wt and K160N depicted as relative light units (RLU) of luciferase reporter activity per µl of pseudovirus stock upon infection of TZM-bl cells. The fold infectivity difference is indicated. (B) Estimates of T for the CAP88 wt and K160N variant. Mean and range of the individual estimates using the R508S/R511S and V513E dominant-negative mutations are shown.

To further explore the relationship between the entry stoichiometry and infectivity we performed additional studies with the subtype C strain CAP88 [43], which had the highest T and lowest infectivity within our panel (Fig. 1B, 1C and 2B). CAP88 is a transmitted/founder virus which carries a lysine (K) at position 160 of gp120, a site frequently targeted by neutralizing antibodies [44]. Among 4894 Env sequences deposited in the Los Alamos HIV Sequence Database asparagine (N) at position 160, as part of an N-linked glycosylation sequon, is with 93.3% the most prevalent residue at position 160 [45]. Loss of this glycosylation site is both associated with escape from PG9/PG16-like antibodies and decreased entry capacity [38], [46]–[48]. Supporting this we found that reconstitution of the N-linked glycosylation site (K160N) in CAP88 results in a 4-fold increase in virus infectivity (Fig. 5A and [38]) highlighting the importance of N160 for env functionality. To probe if the increased infectivity of the CAP88 K160N mutant may be due to changes in trimer structure and function that result in a reduction of T, we analyzed T of CAP88 wt and CAP88 K160N (S8A–S8B Fig.). Indeed, we found that the increased infectivity of CAP88 K160N is reflected by a decreased T (Fig. 5B and S8C Fig.). As this example highlights, changes in trimer structure inferred by naturally occurring mutations, possibly due to antibody escape, can result in a decreased entry capacity of the respective env, which in turn is reflected by an increase in the stoichiometry of entry.

### Entry inhibitor escape mutations can increase the stoichiometry of entry

**Figure 6:**
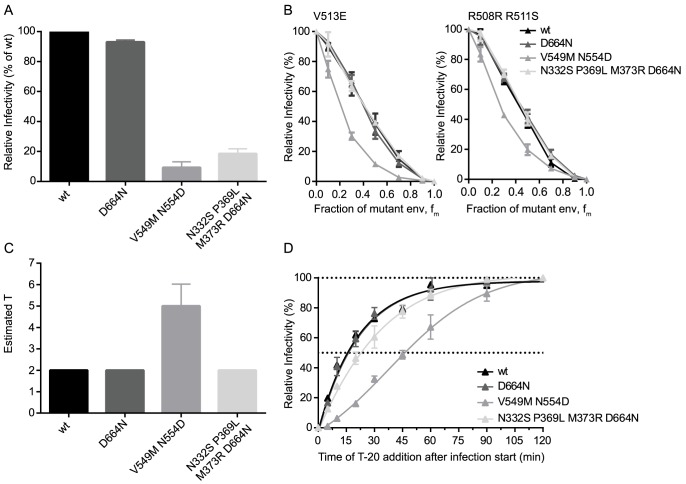
Point mutations in JR-FL dictate virus infection efficacy and entry stoichiometry. (A) Infectivities of JR-FL wt and indicated point mutant envs were determined by titration of virus stocks on TZM-bl reporter cells and are shown normalized to JR-FL wt. Data depict mean and SD from 4 independent experiments. (B) Relative infectivity of mixed trimer infection experiments with the specified JR-FL variants using the R508S/R511S and V513E dominant-negative mutations are shown. Infectivity of pseudotyped virus stocks expressing the indicated ratios of dominant-negative mutant envs was measured on TZM-bl cells. Infectivity of virus stocks containing solely functional envelope were set as 100%. Data depict mean and SD from 2 independent experiments. (C) Mathematical analyses of the data shown in (B) yielded estimates of T, shown here as mean and range of the individual T estimates obtained with the R508S/R511S and V513E dominant-negative mutations. (D) Analysis of virus entry kinetics for the four JR-FL variants were performed as shown S7A Fig.

The finding that a single point mutation in the CAP88 env could dramatically alter entry fitness and entry stoichiometry prompted us to further explore the influence of point mutations on env entry phenotype. To this end we selected three JR-FL variants mimicking resistance mutants as they may occur *in vivo* during neutralization escape: the JR-FL D664N escape mutant resistant to the MPER antibody 2F5, the JR-FL V549M N554D mutant which has a highly increased resistance against the entry inhibitor T-20 [49], and a JR-FL env with point mutations N332S P369L M373R and D664N rendering it resistant against the broadly neutralizing antibodies (bnAbs) PGT128, 2G12, b12 and 2F5 (S9 Fig.). While all three JR-FL variants showed similar mean virion trimer numbers as JR-FL wt, we observed differences in env infectivity with the 2F5 escape mutant infecting equally well as JR-FL wt whereas the T-20 and the multiple bnAb escape mutant env showed strongly reduced infectivity at 9% and 19% of JR-FL wt, respectively (Fig. 6A). When we compared the three escape mutant envs and JR-FL wt in the mixed trimer assays we observed a distinct curve shift for both dominant negative mutants of the T-20 escape variant (Fig. 6B), while the other three envs gave almost identical curves. Mathematical analysis of the data indeed revealed that the T-20 escape mutant requires 4 to 6 trimers for entry while all other mutants, like JR-FL wt, require 2 trimers (Fig. 6C). Hence, the T-20 escape mutant showed both, an increase in T and a loss in infectivity while the bnAb escape mutant env maintained T despite showing infectivity loss, confirming our earlier findings that viruses with the same T can still show a wide variation of infectivities (Fig. 2B). This can possibly be attributed to increased trimer decay rates or variations in CD4 and co-receptor engagement, especially since the bnAb escape mutant carried mutations in the CD4 binding site. Interestingly, the increased demand of the T-20 escape mutant for trimers during entry was also reflected in delayed entry kinetics of this env variant (Fig. 6D) [50]. These entry characteristics of the T-20 escape mutant are intriguing as the resistance mutations lie in the heptad repeat region of gp41 and interfere with six-helix bundle formation, which is a key step providing energy for membrane fusion during the entry process [51]. Thus, it is mechanistically plausible that the mutant env may require more trimers for entry to compensate for the disturbed six-helix bundle formation and generate sufficient energy to achieve membrane fusion.

## Discussion

Fusion of biological membranes, as required for entry of enveloped viruses, occurs in a plethora of cellular processes. In the case of HIV, fusion is executed by envelope glycoprotein trimers upon interaction with adequate receptor molecules on the target cell membrane [1], [8]. While the principle steps are known and thought to be shared across different biological systems and membrane types [7], [52], [53], the exact mechanisms and stoichiometric and thermodynamic requirements of most membrane fusion processes are not completely resolved [1], [8], [10], [11], [54], [55]. Definition of the components involved in HIV entry and the membrane fusion process is of particular interest as improved understanding of the determinants of HIV entry bears the promise to funnel the development of enhanced strategies to prevent and treat viral infections [1], [7]. The efficacy of HIV entry shapes inter- and intra-host transmission and determines the vulnerability to a range of therapeutic and preventive strategies such as neutralizing antibodies, entry inhibitors and antibody based vaccines. Considering that the stoichiometry of entry defines the number of trimers required for a virus to infect, in turn it also defines the number of trimers on a virion that need to be blocked by neutralizing antibodies. Depending on the stoichiometry of entry the quantities of antibody needed for effective neutralization can therefore vary substantially [56] (S10 Fig.).

To resolve molecular requirements of HIV membrane fusion, we explored in the present study the stoichiometry of HIV-1 entry (T), which defines the number of envelope trimers required per virion to fuse with the target cell membrane and thereby initiate infection [13]–[15]. Our estimates of T are based on a combined strategy of experimental data acquisition and mathematical modelling. We analyzed envelopes from 11 HIV-1 strains including different HIV subtypes, CCR5 and CXCR4 users, and envelopes with open (lab adapted strains) and closed (primary isolates) trimer conformation. We found that T differs substantially between individual strains with measurements ranging from 1 to 7 trimers that are required for entry. While a previous study suggested that HIV −1 isolates generally require only a single trimer for entry [13] our analysis retrieved values for T which were, with one exception (strain REJO), greater than 1, supporting the findings of alternate modelling approaches by us and others [14], [15]. Of note, only one of the two dominant-negative env mutants we probed recorded T = 1 for the strain REJO while the other mutant yielded an estimate of T = 2. Thus, while our data cannot exclude that T = 1 for some strains, based on our observations a range of different entry stoichiometries as we describe here seems more plausible.

We postulate two potential underlying causes for the variations in T we observe across strains. Our estimates are based on virion trimer content measurements by ELISA and are therefore a composite of functional and non-functional trimers present on virions. Considering this, a high estimate of T may be derived as the consequence of premature trimer inactivation through rapid trimer decay [38], [57], or a spontaneous adoption of the CD4-bound conformation [57], [58]. In both cases high estimates of T would reflect a decreased proportion of functional trimers on virions. Alternatively, a high T may be required by envelopes which have adopted a trimer conformation with a low energetic state as described for the open conformation of lab adapted strains [58],[59]. There, the lack in energy released upon trimer conformational rearrangements may be compensated by higher trimer numbers roped into the entry process. Most notably, for all three wildtype strains that yielded high estimates of T as well as the V1V2-deleted env variants, the high T was associated with a low infectivity (Fig. 2B, 3A and 3D). The latter is particularly intriguing as it supports the possibility that env deficiencies in entry can be partially overcome by higher numbers of trimers engaged during the entry process. Interesting insights also stem from the SF162/P3N env pair: P3N was isolated from a rhesus macaque after successive rapid transfer following initial challenge with SHIV-SF162 [60]. While SF162 has a high estimate of T of 4 to 5, P3N has a T of 2 and is the most infectious env in our panel (Fig. 2B). Thus, HIV (or SIV) has the potential to evolve from a less fit to a highly transmissible env *in vivo*. The exact mutations responsible for the different phenotypes remain to be determined; as we previously showed, the V1V2 domains of SF162 and P3N appear to play an important role in this regard [38].

A high T was also linked with slower virus entry kinetics (Fig. 4C) suggesting that engagement of multiple trimer-receptor pairings requires prolonged time periods. A similar relationship between kinetics of membrane fusion and the number of involved fusion proteins has been previously demonstrated for SNARE (Soluble NSF Attachment protein Receptor) complex mediated membrane fusion [61], [62] and Influenza virus membrane fusion [16], [17]. The interplay between HIV-1 entry stoichiometry and entry kinetics our study reveals thus underscores the general finding that the kinetics of membrane fusion processes are governed, at least in part, by the number of participating fusion proteins.

We rate the tight association of T with functional properties of the envelopes, namely entry capacity and entry kinetics, as a strong indicator of the validity of our analysis. Nevertheless, such estimates of T can only be an approximation as certain parameters which influence the estimates cannot be determined experimentally and assumptions need to be made for the mathematical analysis. In the literature different approaches towards modelling of mixed trimer experiments have been described and led to partially deviating results, highlighting the importance of validating the models and parameters used [13], [14], [63]. Virion trimer numbers are commonly estimated from gp120 and p24 ELISA data (Table 1) [23], [24]. These analyses yield values for the average envelope content of virions but do not provide information on the frequency distribution of viruses carrying different trimer numbers across a virion population. The latter is a factor that impacts on the interpretation of mixed trimer experiments [14], [15], [64]. Additionally, env content estimates by ELISA do not deliver information on env functionality, hence functional and non-functional trimers will be accounted for [65]. A further potential limitation stems from the nature of the mixed trimer experiments which require that all combinations of envelopes probed lead to a random trimer formation. Since preferential formation of homotrimers could strongly influence results obtained from mixed trimer experiments, we controlled for equal expression levels of the co-expressed env variants. In addition, previous studies from us and others indicate that related env variants indeed form randomly mixed trimers [13], [66], [67]. As outlined in our previous work, we have incorporated in our mathematical model several functions to capture these parameters involved in HIV entry and carefully verified the validity of our approach in the current study both *in vitro* and *in silico* (S1–S2 Fig., S1 Table and [15], [63], [68]). Nevertheless, this does not exclude that additional parameters beyond T contribute to the variation in entry phenotype between individual HIV-1 strains. For instance, differences in trimer stability or affinities for CD4 and co-receptors could significantly impact on virus entry efficacy without direct influence on T.

Membrane fusion via the stalk-pore mechanism [11] is a multi-step process that ultimately depends on energy provided by fusion proteins [9], [11], [52], [69]. Approximation of two membranes is followed by fusion of the two proximal membrane leaflets, forming the hemifusion stalk intermediate. Subsequent fusion of the distal membrane leaflets creates a fusion pore, which may either expand or close again depending on the forces exerted on the membranes. Viral envelope glycoproteins, such as the HIV-1 env or Influenza virus HA trimer, are metastable structures that undergo a series of conformational changes following receptor engagement which releases energy utilized in the fusion process [7], [8]. Although the energy required for hemifusion stalk formation could potentially be recovered from refolding of a single envelope glycoprotein trimer [8], [55], subsequent formation of the fusion pore and pore enlargement are thought to require higher energy levels [9], [11], [70]. Growing evidence suggest that only concerted action of several trimers leads to membrane fusion and maintenance of a fusion pore large enough to allow passage of the HIV capsid [1], [9], [10], [20]. Our estimates that, for the majority of HIV-1 primary isolates, 2 to 3 env trimers are required to mediate infection are thus in accordance with these studies on the mechanisms of membrane fusion. Interestingly, our estimates of the HIV entry stoichiometry resemble those made for Influenza A virus [16]–[18], postulated to require 3 to 4 HA trimers for entry, and vesicular membrane fusion via SNARE complexes, which generate energy during refolding at levels comparable to viral fusion proteins [71]–[73]. In high similarity to HIV and Influenza, also 1 to 3 SNARE complexes appear to be required to induce membrane fusion [61], [62], [74]. To further explore this relationship, we compared reported values of energy required for membrane fusion and energies released by fusion proteins (S11 Fig.). Membrane fusion requires an energy input of 40 to 120 k_b_T [9], [55], [75], [76]. Refolding of HA trimers into the six-helix bundle conformation was estimated to release 30 to 60 k_b_T [8], [9], [77] while SNARE complex assembly into 4-helix bundles releases an estimated 19 to 65 k_b_T [71]–[73], [78]. Considering that 3 to 4 HAs and 1 to 3 SNARE complexes were estimated to participate in entry, this yields total energies of 20 to 240 k_b_T released during the respective membrane fusion processes. In analogy, assuming a total energy of 40 to 120 k_b_T required for membrane fusion, our estimates that 2 to 7 trimers are required for HIV entry indicate that each trimer releases between 6 to 60 k_b_T during the entry process. Of note, the calculated total energies released by both HA trimers and SNARE complexes appear to be higher than the reported energy required for membrane fusion. This could potentially be due to inefficient coupling of the energy generated through protein conformational rearrangements into membrane deformation, or divergence between artificial membranes employed in biophysical experiments to measure membrane fusion energies and naturally occurring membranes containing proteins and having varying lipid composition. In sum, the strong agreement in the estimated energies across biological systems is intriguing and suggests that the overall energy requirements of membrane fusion and principles of energy elicitation by fusion proteins are closely related.

Our estimates that HIV strains typically require 2 to 7 trimers for entry are also consistent with the observations that env trimers cluster on virions and that HIV establishes a contact zone where several env-receptor pairings between virus and target cell are formed, the so-called “entry claw” [19], [28]. When defining molecular requirements for HIV entry, it is important to consider that envelope trimer activities may reach beyond solely providing the energy for membrane fusion. For example, binding of HIV trimers to their target cell receptors triggers an array of intracellular signals, which amongst other processes is thought to govern intracellular actin re-arrangements [79] and may be required to support membrane fusion and pore enlargement as previously proposed [10], [11].

In summary, our estimates of the HIV entry stoichiometry are in strong accordance with requirements found in other membrane fusion processes. Importantly, we show that capacities of individual HIV envelopes in mediating entry can vary substantially which is likely due to differences between trimers in soliciting energy required for membrane fusion. Our data strongly suggest that viruses overcome these envelope limitations by increasing the number of envelope-receptor pairings involved in the entry process. Hence, the stoichiometry of HIV entry is an important parameter steering virion infectivity and its assessment provides a relevant contribution towards a refined understanding of HIV-1 entry and pathogenesis. Knowledge obtained from the quantitative assessment of trimer-receptor interactions during HIV entry is a prerequisite in unravelling the stoichiometry of trimer interactions with target cell receptors or virus inactivation by neutralizing antibodies and entry inhibitors and thus may aid future approaches in HIV vaccine or entry inhibitor design.

## Materials and Methods

### Cells, viruses and inhibitors

293-T cells were obtained from the American Type Culture Collection (ATCC) and TZM-bl cells [80] from the NIH AIDS Research and Reference Reagent Program (NIH ARP). Both cell types were cultivated in DMEM (Gibco) containing 10% heat inactivated FCS and penicillin/streptomycin. Plasmids encoding the envelopes of strains JR-FL, SF162, NL4-3, RHPA, AC10, REJO, BG505 and ZM214 were obtained from the NIH ARP. Envelope ZA110 was described previously [67]. Envelope clone P3N [60] was a gift from Dr. Cecilia Cheng-Mayer, Aaron Diamond AIDS Research Center, New York, USA. Envelope clone CAP88 [81] was a gift from Dr. Lynn Morris, National Institute for Communicable Diseases, Johannesburg, South Africa. All envelope point mutations were generated by site-directed mutagenesis (Agilent QuikChange II XL) according to the manufacturer instructions. All point mutant envelopes were sequenced by in-house Sanger sequencing to confirm presence of the desired mutations and absence of unintended sequence changes. V1V2-deleted envelopes were previously described [67]. The Luciferase reporter HIV pseudotyping vector pNLLuc-AM was previously described [67]. T-20 [82] was purchased from Roche Pharmaceuticals.

### Estimating the stoichiometry of virus entry by mixed trimer experiments

To estimate the stoichiometry of entry we employed a previously described approach [13]. To produce HIV-1 pseudotype virus stocks expressing mixed trimers with varying ratios of functional to dominant-negative env, 293-T cells in 12 well plates (100.000 cells per well in 1 ml complete DMEM, seeded 24 h pre-transfection) were transfected with 1.5 µg pNLLuc-AM and 0.5 µg env expression plasmids, using polyethyleneimine (PEI) as transfection reagent. The ratio of functional to dominant negative env expression plasmids was varied to yield combinations with 100, 90, 70, 50, 30, 10 and 0% of functional env. Total env plasmid content was always at 0.5 µg. After overnight incubation the transfection medium was replaced with 1 ml fresh complete DMEM and virus-containing supernatants were harvested 48 h post transfection. All mixed trimer combinations of an individual virus strain were always generated in parallel, excluding influences of producer cells and transfection procedure. To determine virus infectivity, serial dilutions of virus stocks starting with 100 µl of undiluted virus supernatant were added to TZM-bl reporter cells in 96-well plates (10.000 cells per well) in DMEM medium supplemented with 10 µg/ml DEAE-Dextran. TZM-bl infection was quantified 48 h post-infection by measuring activity of the firefly luciferase reporter. For each functional to dominant negative env ratio series, the infectivity of the stock containing 100% functional (wt) env was taken as reference (100% infectivity) and the relative infectivity of the stocks with increasing percentages of dominant-negative env were calculated in relation to that infectivity value. The resulting data of relative infectivity were plotted over the fraction of dominant-negative env of each stock and the data were analyzed with mathematical models [15] as described below.

### Mathematical modeling of entry stoichiometry data

#### Mathematical model

Previously, we described mathematical models to analyze relative infectivity data generated with virions expressing mixed trimers [15]. Starting point of the mathematical modeling was a basic model, in which we made the following assumptions concerning the experimental system:

- During the transfection of virus producer cells, the fraction of plasmids encoding wild-type, f_wt_, and mutant, f_m_, envelope proteins are identical inside and outside the cells, and the pool of produced envelope proteins inside the transfected cell reflects these fractions as well.
- Envelope proteins assemble randomly into trimers. More precisely, the number of mutant envelope proteins in a trimer follows a Binomial-distribution with a trial parameter 3 and a success parameter f_m_.
- Trimers can move freely on the viral surface, i.e. each functional trimer can potentially engage with cellular receptors to assist in mediating cell entry.

To verify the use of this model for our data analysis we assessed the validity of the underlying assumptions listed above. Pseudotyped virions are produced by transfecting cells with different plasmids encoding for env and the required structural and accessory HIV proteins. This co-transfection approach and the existence of heterotrimers in setups with plasmids encoding for different envelope proteins show that more than one plasmid can enter a virus producer cell. Thus, we conclude that sufficient numbers of plasmids enter transfected cells to guarantee that the envelope pool composition reflects the composition of plasmids encoding for the different envelope variants, i.e. assumption (i) is justified. Previous studies from others and us indicated that related env variants form randomly mixed trimers [13], [66], [67], [83], i.e. assumption (ii) is justified. Chojnacki et al. [28]showed that trimers on mature virions can move freely, i.e. assumption (iii) is also justified.

The model predicts the relative infectivity of a virion stock produced with a given fraction of mutant envelope proteins f_m_, the fraction of virions with s trimers, η_s_ 0≤s≤s_max_, and the stoichiometry of entry, T:
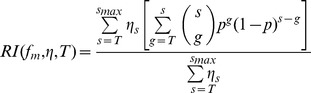
where *p* = (1−*f_m_*)^3^ is the probability that a trimer consist of only wild-type envelope proteins and is therefore functional. s_max_ is the maximal number of trimers on a virion's surface. In the numerator we calculate the probability that a virion with s trimers can infect a cell, i.e. has at least T functional trimers (i.e. the expression in the squared brackets). This probability is weighted by the probability that a random virion has s trimers, η_s_. All these probabilities are summed up for virions having at least T trimers. This expression is then divided by the probability that a random virion expressing only wildtype envelope proteins is infectious, because we normalize the measured infectivities accordingly in the experiments. For additional analyses of the relative infectivities we also employed model extensions described earlier [15]. In two model extensions we relaxed one of the assumptions (i) or (ii) as defined above and in a third model extension we implement an incremental increase of virus infectivity with trimer numbers. For a more detailed derivation of the RI-predictions predictions in the basic model and the different extensions please refer to [15].

#### Trimer number distributions

The trimer number distribution is an important input parameter and the relative infectivity prediction is sensitive to changes in the trimer number distribution [15]. In our previous work, we based our estimations of the parameter T on discretized B-distributed trimer numbers with mean 14 and variance 49 as reported by counting trimers on the virion surface in cryo-electron microscopy experiments [26]. In the present study, we experimentally determined the mean trimer numbers of the 11 different viral strains included in our study (Table 1). However, our measurements do not allow inferring higher moments of the distribution of the trimer numbers, such as the variance. To overcome this problem, we calculated the trimer number distributions using the discretized B-distribution defined in [15] with the measured mean trimer numbers, μ (Table 1), and the variance calculated according to v = 49/14*μ [26]. The maximal trimer number, s_max_, was set to 100.

#### Analysis

To estimate T, we fitted the relative infectivity function to experimentally obtained relative infectivity values by assuming trimer number distributions calculated as described above applying a non-linear regression algorithm. Because relative infectivity data ranges from 0 to 1, the data was transformed with the arcsin-sqrt function. To gain an accuracy measure of our estimates, we perform a bootstrap procedure with 1000 replicates. All analyses were performed using the statistical software package R [84]. The corresponding code is available upon request.

### Production of pseudotype virus stocks for infectivity assessment and entry kinetics

To produce HIV-1 pseudotype virus stocks for comparisons of virus infectivity and determination of virus entry kinetics, 293-T cells in T75 flasks (2.250.000 cells in 15 ml complete DMEM, seeded 24 h pre-transfection) were transfected with env and pNLLuc-AM plasmids (7.5 µg and 22.5 µg, respectively) using PEI as transfection reagent. The medium was exchanged 12 h post-transfection and virus-containing supernatants were harvested 48 h post-transfection. The supernatants were cleared by low speed centrifugation (300 g, 3 minutes), aliquoted and stored at −80°C. Virus infectivity was quantified on TZM-bl reporter cells as described above.

### gp120 and p24 ELISA of pseudotype virus stocks

To determine gp120 and p24 content of HIV-1 pseudotype virus preparations, 293-T cells in T25 flasks (750.000 cells in 5 ml complete DMEM, seeded 24 h pre-transfection) were transfected with env and pNLLuc-AM plasmids (2.5 µg and 7.5 µg, respectively) using PEI as transfection reagent. The medium was exchanged 12 h post-transfection and virus-containing supernatants were harvested 48 h post-transfection. The supernatants were cleared by low speed centrifugation (300 g, 3 minutes), then ultracentrifugated (SW28 rotor, 2 h, 28.000 rpm, 4°C), the supernatant removed and viral pellets resuspended in 0.3 ml cold PBS and stored at −80°C. Virion associated p24 and gp120 antigens were quantified by ELISA as previously described [67]. Briefly, virus preparations were dissolved in 1% Empigen (Fluka Analytical, Buchs, Switzerland) and dilutions of each sample probed for gp120 and p24. Gp120 was captured on anti-gp120 D7324 (Aalto Bioreagents, Dublin, Ireland) coated immunosorbent plates and detected with biotinylated CD4-IgG2 and Streptavidin-coupled Alkaline Phosphatase (GE Healthcare, Chalfont St Giles, UK). P24 was captured on anti-p24 D7320 (Aalto Bioreagents, Dublin, Ireland) coated plated and detected using Alkaline Phosphatase-coupled antibody BC1071 (Aalto Bioreagents, Dublin, Ireland). Concentrations of gp120 and p24 in the samples were calculated in relation to standard curves obtained with gp120 and p24 proteins of known concentration. To derive virion numbers from p24 concentrations, we assumed 2000 (for the data in main manuscript and figures) or alternatively 2400 (S3B Fig.) p24 molecules per virion [23], [24], [33]. Trimers per virion were then calculated from the obtained gp120 concentration in relation to the number of virions per sample.

### Virus entry kinetics

To determine virus entry kinetics, TZM-bl cells were seeded in 96-well plates (20.000 cells per well) in complete DMEM supplemented with 10 µg/ml DEAE-Dextran. 24 h post-seeding, cells were first cooled at 4°C for 5 minutes, then the medium was removed. HIV-1 pseudotype virus stocks adjusted to 50.000 RLU in 100 µl DMEM at 4°C were added per well and plates centrifuged for 70 minutes at 1200 g and 10°C. The low temperature was chosen to allow virus attachment during spinoculation but no entry. Following spinoculation the supernatant with unbound virus was removed and 130 µl of DMEM, pre-warmed to 37°C, were added per well to initiate infection (timepoint zero) and plates were incubated at 37°C. At defined timepoints post-infection, 20 µl of T-20 (375 µg/ml in DMEM; yielding a final assay concentration of 50 µg/ml) were added per well to stop the viral entry process. To obtain a measure for infectivity across different experiments, the wells with the last T-20 addition at 120 min after infection start were used as 100% reference infectivity value and the infectivity of all other T-20 treated wells were set in relation to it. In addition, a mock-treated well (addition of 20 µl DMEM at timepoint zero) was evaluated to assess absolute infectivity in absence of T-20.

### PBMC infection experiments

Isolation and infection of PBMCs was performed as previously described [85]. Briefly, PBMCs were isolated from pooled buffy coat of 3 healthy blood donors, stimulated with anti-CD3 and PHA in presence of 100 U IL-2 for 2 days and then seeded in 96-well plates at a density of 100.000 cells per well in 100 µl RPMI medium supplemented with 10% FCS, antibiotics, 100 U IL-2 and 2.5 µg/ml (final assay concentration) polybrene. Serial dilutions of mixed trimer virus stocks (100 µl per well) were added to the PBMCs and cells were incubated at 37°C for 72 h before determining luciferase reporter activity.

### Statistical analyses

Correlation analyses according to Pearson and multiple unpaired t-tests to derive the p-values for the comparisons of wt and V1V2-deleted envs were performed in GraphPad Prism 6.

## Supporting Information

S1 Fig **Model curve fits and verification of T estimates by bootstrap analyses.** The graphs depict for each virus strain the empirical data shown in Fig. 1B and the according curve fits obtained with our model (“basic model”). The dominant negative mutants are shown in black (R508S/R511S) and red (V513E) respectively. The insets show the results of a bootstrap analysis with 1000 replicates as a measure of accuracy of our best fit estimate (marked with an asterisk).
(PDF)

S2 Fig
**Model extensions for strain CAP88.** (A) to (D): To verify if the basic model we used for data analysis is indeed the best choice we performed three extensions of our model and show the results here using strain CAP88 as an example; details of the model fits for all 11 strains are listed in S1 Table. Graphs depict the empirical data of CAP88 shown in Fig. 1B and the according curve fits obtained with our model. The dominant negative mutants are shown in black (R508S/R511S) and red (V513E) respectively. (A) Fit of the basic model, as shown in S1 Fig. (B) Fit of the imperfect transfection model which simulates incomplete transfection of producer cells with env plasmids during production of mixed trimer virus stocks. With this model we estimate both T and the coefficient of variation (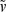). The coefficient of variation ranges from 0 to 1 and is a measure of how different the mixture of envelope proteins inside the transfected cell is in comparison to the env plasmid mixture used to transfect the cell. 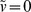 corresponds to a perfect match (thus this is mathematically equal to the basic model), 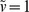 correspond to cells producing only one type of envelope protein. Note that we obtain very different estimates both for T and the coefficient of variation despite very similar empirical data for the two mutants. The insets show the accuracy of the estimates determined in a bootstrap procedure with 1000 replicates (each gray point represents one bootstrap replicate); the colored dot shows the estimated values of the best fit. (C) Fit of the segregation model, simulating preferential segregation of the wt and mutant envs produced in a transfected cells into homotrimers. From this model we estimate both T and a parameter for the magnitude of the segregation (*ξ*), ranging from 0 to 1. *ξ* = 0 corresponds to perfectly randomly mixed trimers (equal to the basic model) and *ξ* = 1 corresponds to formation of wt and mutant homotrimers only. We obtain very high estimates of T as well as a segregation parameter close to or equal to 1. The insets show the results of 1000 bootstrap replicates (gray points) and the best fit parameters (colored points). (D) Fit of the soft threshold/incremental model, where virus infectivity scales with the trimer number, s, according to the equation 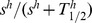. From this model we estimate the trimer number for which 50% infectivity is reached, T_1/2_, and the steepness of the infectivity increase (h). In our analysis T_1/2_ cannot be bigger than 100. The insets show the results of 1000 bootstrap replicates (gray points) and the best fit parameters (colored points).(PDF)

S3 Fig **Extended analyses for T assuming different mean virion trimer contents.** (A) To probe the influence of the mean virion trimer content on the estimates of T we re-analyzed the data shown in Fig. 1B. Here, instead of the measured individual mean trimer numbers of each strain (Table 1), identical mean trimer numbers for all 11 strains were assumed. We chose 3 values for this comparison that covered the range of trimer numbers measured across our panel: mean trimer numbers of 5 and 26 (representing the lowest and highest trimer contents measured in individual experiments) and a mean trimer number of 13, the mean of trimer numbers measured across our virus panel. Data depict the resulting estimates of T as mean and the range of the independent estimates for both the R508S/R511S and V513E mutations. Applying these fixed trimer numbers instead of the individually measured values to our dataset we obtained for the low mean trimer number of 5 the lowest estimates of T ranging from 1 to 5 trimers. For a mean trimer number of 26, T ranged between 3 and 17 trimers and for the mean trimer number 13, T ranged from 1 to 10 trimers. (B) In an additional analysis we performed a correction of the measured mean virion trimer numbers shown in Table 1 by assuming 2400 p24 per virion instead of 2000 p24. This results in a 20% increase in mean virion trimer numbers. Incorporating these higher mean virion trimer numbers in our analysis yielded the estimates of T shown here, which are identical or slightly higher than the estimates obtained with the original mean trimer numbers. While these comparisons confirm that absolute numbers of T estimates can vary if there are fluctuations in mean trimer numbers of a virus, the qualitative differences in T amongst viruses persisted, highlighting that they reflect qualitative entry properties of the respective virus envelopes and less their quantitative expression.
(PDF)

S4 Fig **Comparison of TZM-bl and PBMC as target cells in mixed trimer experiments.** (A) and (B): Mixed trimer virus stocks of strains JR-FL and SF162 carrying the R508S/R511S dominant negative mutation were assayed on TZM-bl reporter cells and healthy donor PBMC. In both cases, the obtained PBMC curves closely match the TZM-bl data (see also Fig. 1 D and E). The resulting estimates of T are identical for JR-FL and deviate by one trimer for SF162. In (C) and (D) we show bootstrap analyses with 1000 replicates for the SF162 data, indicating that the actual difference between the estimated T's is small as in both cases T = 4 or 5 are the two most frequent estimates. This indicates that our approach to estimate T is independent of target cells used and yields results that are physiologically relevant. Data depict mean and SD from 2 independent experiments.
(PDF)

S5 Fig **Influence of V1V2-deleted virion gp120 content on estimates of T.** Virion gp120 content of V1V2-deleted envs was experimentally determined and compared to wt virion gp120 content. Black bars depict the logarithmic fold difference between V1V2 and wt env expression. Values are means of 2 to 3 independent experiments. The V1V2-deleted envs proved to be expressed and incorporated into virions at similar levels as the wt and were all in the range of 80 to 100% of the corresponding wt envs. The actual V1V2-deleted virion mean trimer contents were then employed in the estimation of T as shown in Fig. 3D. In addition, to control for influences of env expression on our estimates of T, we calculated for each wt – ΔV1V2 env pair the required env content differences that theoretically would be required to cause the observed curve shifts for V1V2-deleted virions in the mixed trimer experiments (Fig. 3B and C), if wt and ΔV1V2 strains would have identical T's. These calculated differences in env expression are shown for the R508S/R511S (green bars) and V513E (red bars) mutations, respectively. While env content certainly influences the estimation of T, we found that only substantial env content differences (between 30 to more than 90% lower than wt for the majority of strains) would result in the observed differences between wt and V1V2-deleted envs. Hence, even for those viruses where expression of the V1V2 deleted env was lower than the wt in our experiments (black bars), this loss proofed not sufficient to induce the shifts in T we observed (Fig. 3D). Thus these analyses confirm that the loss in entry efficiency upon V1V2 deletion we observe is not simply caused by lower envelope content of these virions but indeed indicates a higher T.
(PDF)

S6 Fig **Influence of increased virion trimer numbers on estimates of T and virus population infectivity.** (A–C) To further probe the influence of trimer content on T we compared estimates of T and infectivity of viruses on which env content was modulated. According to our model, the amount of functional trimers per virion, in combination with the number of trimers required for entry (T), is a decisive parameter for virion infectivity. (A and B) We produced mutants of JR-FL wt and JR-FL ΔV1V2 with a deletion of the gp41 cytoplasmic tail (CT) by replacing gp41 tyrosine 712 with a STOP codon. (A) 293-T cells were transfected with env encoding plasmids and gp120 expression was determined by cell surface staining with anti-gp120 MAb 2G12 and flow cytometry. Data depict one of two representative experiments. (B) gp120 content measurements for wt and CT deleted JR-FL wt and JR-FL ΔV1V2 were performed by gp120 ELISA. Bars show increase in gp120 content of CT truncated envs compared to full length envelopes. Mean and SD of two independent experiments are shown. As described [39], [40] removal of the endocytosis signal in the CT domain resulted in a higher gp120 content on the surface of JR-FL env-expressing cells and on pseudotyped virions (1.5 and 2-fold higher gp120 content, for JR-FL wt and JR-FL ΔV1V2, respectively. (C) Infectivity of wt and CT deleted virus stocks was determined by titration of virus supernatants on TZM-bl cells and was recorded as activity of the luciferase reporter (relative light unit; RLU) per µl virus stock. Data are mean and SD from 2 independent experiments. The fold change in infectivity upon CT deletion is indicated above the bars for both wt and V1V2 deleted envs. CT-deleted JR-FL wt env infectivity increased by 2.1-fold compared to full-length JR-FL wt, while infectivity of the CT-deleted ΔV1V2 env was elevated 9.4-fold compared to the CT-containing ΔV1V2 env. Thus, the ΔV1V2 env, which has a higher T than the wt env (Fig. 3D), appears to profit more from increased trimer numbers than the wt env, supporting the hypothesis that the interplay between virion trimer numbers and T is a major parameter determining virion infectivity. (D and E) Diagrams illustrating the influence of trimer content on the infectivity of a virus population using the example of the observed effect of env overexpression by CT deletion on JR-FL wt (D) and ΔV1V2 envs (E). As shown in Fig. 3D, we observed a higher T for JR-FL ΔV1V2 than for JR-FL wt env. Let us assume that the distribution of trimer numbers across a population of JR-FL wt and ΔV1V2 virions is similar and follows a Gaussian distribution. Then, less virions of JR-FL ΔV1V2 will have the required number of trimers (T_ΔV1V2_) than wt virions (T_wt_) to be infectious (for both virion populations, only virions on the right side of the T_wt_ or T_ΔV1V2_ threshold are assumed to be infectious). Thus, the infectivity of the JR-FL ΔV1V2 population is lower than JR-FL wt. Upon CT deletion, the number of trimers per virion will increase for both the JR-FL wt and ΔV1V2 population. For JR-FL wt, the resulting gain in population infectivity is rather modest (2-fold as shown in panel C), because, as indicated in (D), the majority of JR-FL wt virions already has the required number of trimers to be infectious and the additional proportion of virions that shifts over the T_wt_ threshold is rather small. However, for JR-FL ΔV1V2 virions CT deletion will result in a large proportion of virions shifting over the T_ΔV1V2_ threshold (E), thereby explaining the larger increase in ΔV1V2 virion population infectivity seen upon CT deletion (9.4-fold as shown in panel C). Thus, we conclude that the CT deletion data support our findings of different Ts between wt and V1V2-deleted envs and underline the interplay between virion trimer content and T that governs virion infectivity.
(PDF)

S7 Fig **Determination of virus entry kinetics.** (A) Scheme of the time-of-inhibitor addition experiment employed to determine virus entry kinetics. In this experiment synchronized infection of TZM-bl cells with pseudoviruses was terminated at consecutive time points by the fusion inhibitor T-20. (B) Virus entry kinetics for the ten pairs of wt and V1V2-deleted strains are depicted as relative infectivity over time of T-20 addition. Infectivity was measured following pseudovirus infection of TZM-bl cells by recording firefly luciferase reporter activity. Infectivity reached after 120 minutes was set as 100% and all data were normalized relative to this value. Data are mean and SD from 2 to 4 independent experiments. From the depicted data, time required for each strain to reach 50% of entry as shown in Fig. 4B of the main text are shown.
(PDF)

S8 Fig **Estimation of T for the CAP88 K160N envelope variant.** Relative infectivity of mixed trimer infection experiments of CAP88 wt and the K160N variant using the R508S/R511S (A) and the V513E (B) dominant-negative mutations are shown. Infectivity of pseudotyped virus stocks expressing the indicated ratios of dominant-negative mutant envs was measured on TZM-bl cells. Infectivity of virus stocks containing solely wt envelope were set as 100%. Data depict mean and SD from 2 independent experiments. (C) Estimates of T based on the data depicted in (A) and (B) were derived from 4 different analyses in which we included either the experimentally derived CAP88 mean virion trimer content (identical for both wt and K160N) or assumed trimer contents of 5, 13 and 26 trimers per virion (see also S3A Fig.). Data points depict mean and range of the independent estimates for the R508S/R511S and V513E mutations.
(PDF)

S9 Fig **Neutralization escape of JR-FL point mutant envs.** (A) to (C): Neutralization of virus by inhibitors was determined by incubating virus stocks with serial dilutions of inhibitors at 37°C for 1h. Subsequently, the virus-inhibitor mixes were transferred to TZM-bl reporter cells and infection was quantified 48h later by luciferase reporter read-out. Infection of cells with mock-treated virus was set to 100% of infectivity (0% neutralization) and % neutralization of virus incubated with different concentrations of inhibitors was calculated in relation to that value. Data depict mean and SD from 2 to 3 independent experiments. (A) Neutralization of JR-FL wt and JR-FL D664N by antibody 2F5. (B) Neutralization of JR-FL wt and JR-FL V549M N554D by T-20. Note that the mutant is still sufficiently sensitive to T-20 to allow assessment of entry kinetics as shown in Fig. 4D and S7A Fig. (C) Neutralization of JR-FL wt and JR-FL N332S P369L M373R D664N by antibodies PGT128, 2G12, b12 and 2F5.
(PDF)

S10 Fig **Relation of entry stoichiometry and number of antibodies required for neutralization.** Scheme depicting how the stoichiometry of entry influences the efficacy of HIV virion population neutralization by antibodies. Let us assume two virions, one with a stoichiometry of entry of 2, and one with a stoichiometry of entry of 7 (according to Fig. 1C). Both virions carry 13 trimers, and in both cases binding of 1 antibody per trimer is assumed to be sufficient to block trimer functionality. Thus, a minimum of 12 antibodies is required to neutralize the virion with T = 2, while only 7 antibodies are required to neutralize the virion with T = 7, representing a 71% difference in antibody numbers required for neutralization. On a virus population level, these differences in entry stoichiometry may substantially influence the efficacy of neutralizing antibodies [56].
(PDF)

S11 Fig **Summary of energies required for membrane fusion and provided by fusion proteins.** Values reported in the literature (see main text for details) of the energies required for biological membrane fusion or released by Influenza HA trimers and vesicular SNARE complexes during refolding into the post-fusion conformation are shown. Based on the reported stoichiometries of Influenza virus entry and SNARE-mediated membrane fusion we calculated the total energy contributed by these fusion proteins during the membrane fusion process. In analogy, assuming 40 to 120 K_b_T for membrane fusion and that 2 to 7 HIV-1 env trimers participate in entry, we derive that each env trimer needs to contribute 6 to 60 K_b_T during refolding into the post-fusion conformation to mediate membrane fusion. K_b_ is the Boltzmann constant.
(PDF)

S1 Table **Parameters and estimated T values obtained with model extensions.** We tested three extensions of our mathematical model (referred to here as “basic model”) to analyze the empirical data shown in Fig. 1B. These extensions include a model accounting for imperfect transfection, a model accounting for envelope protein segregation and a model where we relax the assumption of an absolute threshold for T (soft threshold model). For the first three models we show the estimated T values, as well as the coefficient of variation (imperfect transfection model) and a parameter for segregation (segregation model). For the soft threshold model we show the estimated T_1/2_, which is the trimer number where 50% infectivity is reached, and the parameter h defining the steepness of the infectivity increase. For all three model extensions we determined the improvement of the fit compared to the basic model with an F-test and show the corresponding p-value as a measure of significance (p<0.05 means significant improvement; see also S2 Fig. for details of the alternative model fits.) These significantly better fits are not necessarily an indication of biologically more meaningful results. Suitability of model predictions need to be decided on by taking the question being addressed into account and whether the estimated parameters are in accordance with the biological system investigated. In our case, all models except the basic model yielded in several cases T estimates that are far higher than what can be expected in the context of HIV infection. In addition, the basic model was the only one that yielded comparable T estimates for both dominant negative mutants in the majority of the probed strains.
(PDF)
