# Supplementary figures and images for: Different Infectivity of HIV-1 Strains Is Linked to Number of Envelope Trimers Required for Entry
Source: PLoS Pathog. 2015 Jan 8;11(1):e1004595. doi: 10.1371/journal.ppat.1004595 (PMC4287578; doi:10.1371/journal.ppat.1004595)

Supplementary Figure S1

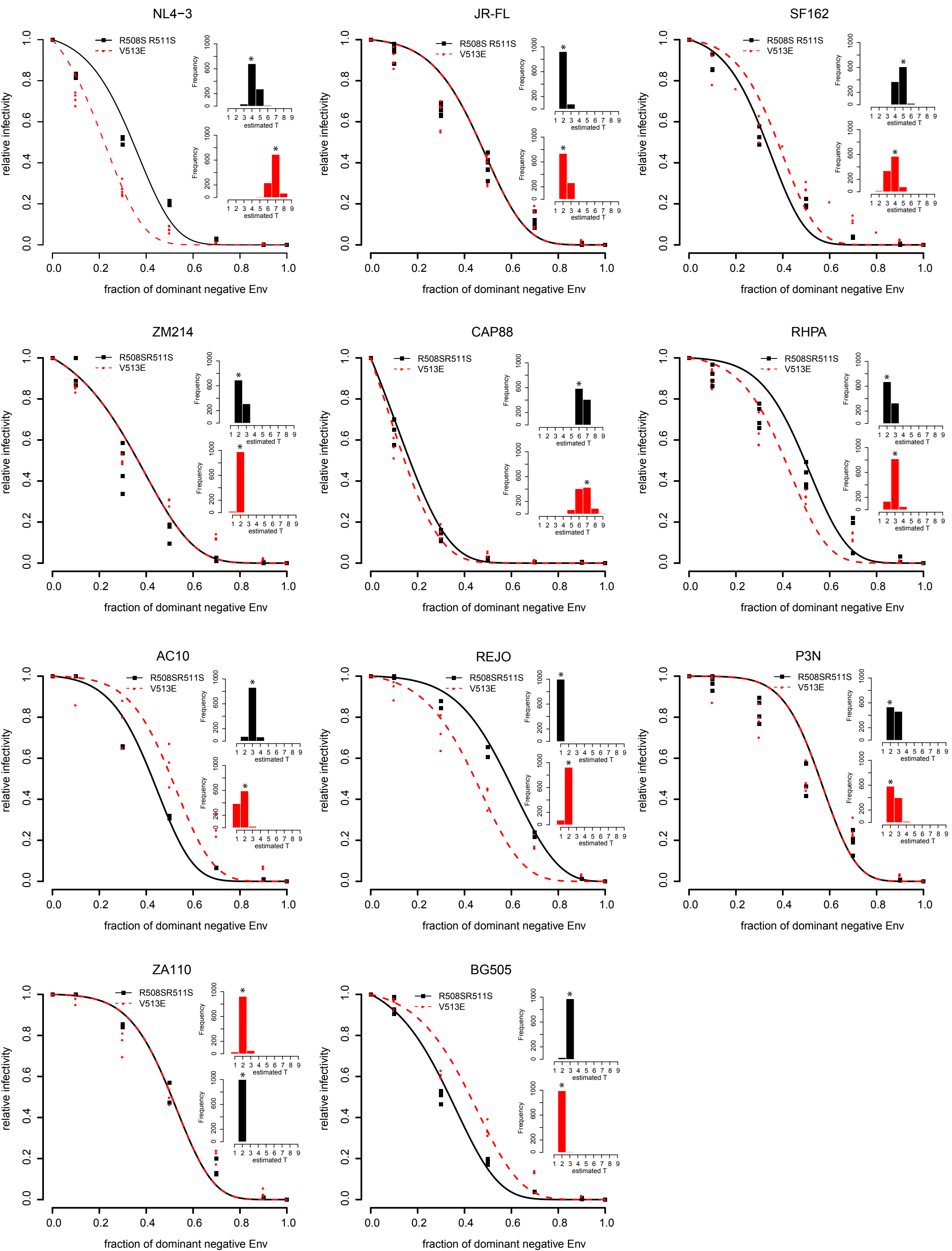

Supplement: S1 Fig — Model curve fits and verification of T estimates by bootstrap analyses. The graphs depict for each virus strain the empirical data shown in Fig. 1B and the according curve fits obtained with our model (“basic model”). The dominant negative mutants are shown in black (R508S/R511S) and red (V513E) respectively. The insets show the results of a bootstrap analysis with 1000 replicates as a measure of accuracy of our best fit estimate (marked with an asterisk). (PDF) [file ppat.1004595.s001.pdf]

# Supplementary Figure S3

A

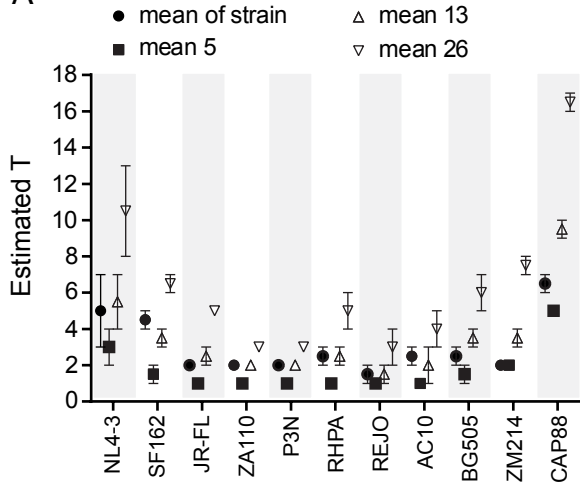

B

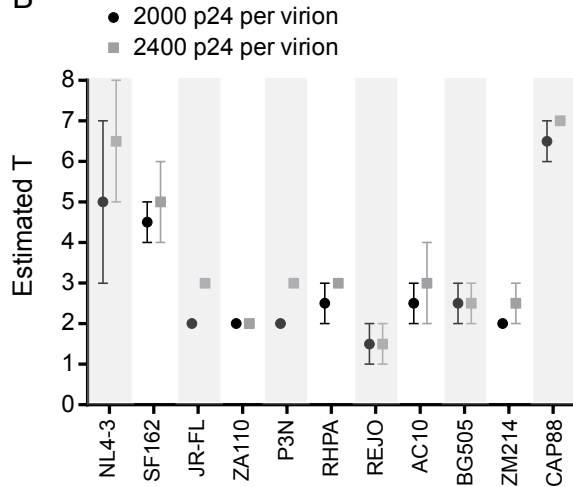

Supplement: S3 Fig — Extended analyses for T assuming different mean virion trimer contents. (A) To probe the influence of the mean virion trimer content on the estimates of T we re-analyzed the data shown in Fig. 1B. Here, instead of the measured individual mean trimer numbers of each strain (Table 1), identical mean trimer numbers for all 11 strains were assumed. We chose 3 values for this comparison that covered the range of trimer numbers measured across our panel: mean trimer numbers of 5 and 26 (representing the lowest and highest trimer contents measured in individual experiments) and a mean trimer number of 13, the mean of trimer numbers measured across our virus panel. Data depict the resulting estimates of T as mean and the range of the independent estimates for both the R508S/R511S and V513E mutations. Applying these fixed trimer numbers instead of the individually measured values to our dataset we obtained for the low mean trimer number of 5 the lowest estimates of T ranging from 1 to 5 trimers. For a mean trimer number of 26, T ranged between 3 and 17 trimers and for the mean trimer number 13, T ranged from 1 to 10 trimers. (B) In an additional analysis we performed a correction of the measured mean virion trimer numbers shown in Table 1 by assuming 2400 p24 per virion instead of 2000 p24. This results in a 20% increase in mean virion trimer numbers. Incorporating these higher mean virion trimer numbers in our analysis yielded the estimates of T shown here, which are identical or slightly higher than the estimates obtained with the original mean trimer numbers. While these comparisons confirm that absolute numbers of T estimates can vary if there are fluctuations in mean trimer numbers of a virus, the qualitative differences in T amongst viruses persisted, highlighting that they reflect qualitative entry properties of the respective virus envelopes and less their quantitative expression. (PDF) [file ppat.1004595.s003.pdf]

Supplemental Figure S4

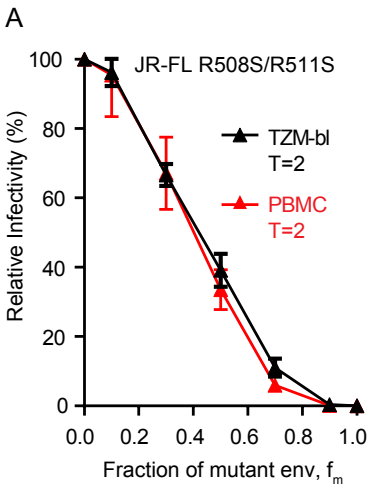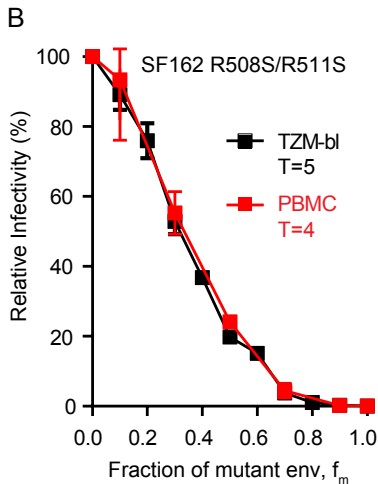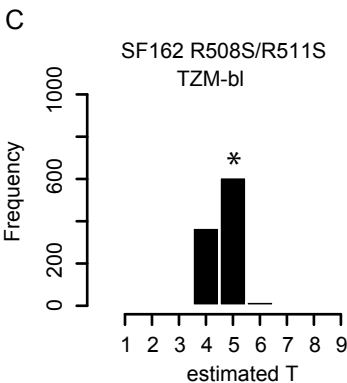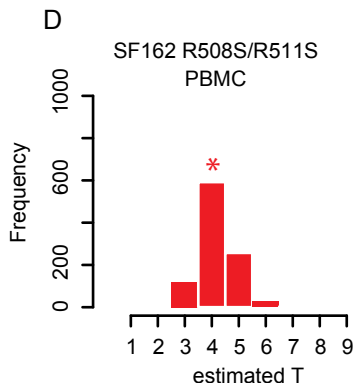

Supplement: S4 Fig — Comparison of TZM-bl and PBMC as target cells in mixed trimer experiments. (A) and (B): Mixed trimer virus stocks of strains JR-FL and SF162 carrying the R508S/R511S dominant negative mutation were assayed on TZM-bl reporter cells and healthy donor PBMC. In both cases, the obtained PBMC curves closely match the TZM-bl data (see also Fig. 1 D and E). The resulting estimates of T are identical for JR-FL and deviate by one trimer for SF162. In (C) and (D) we show bootstrap analyses with 1000 replicates for the SF162 data, indicating that the actual difference between the estimated T's is small as in both cases T = 4 or 5 are the two most frequent estimates. This indicates that our approach to estimate T is independent of target cells used and yields results that are physiologically relevant. Data depict mean and SD from 2 independent experiments. (PDF) [file ppat.1004595.s004.pdf]

Supplementary Figure S5

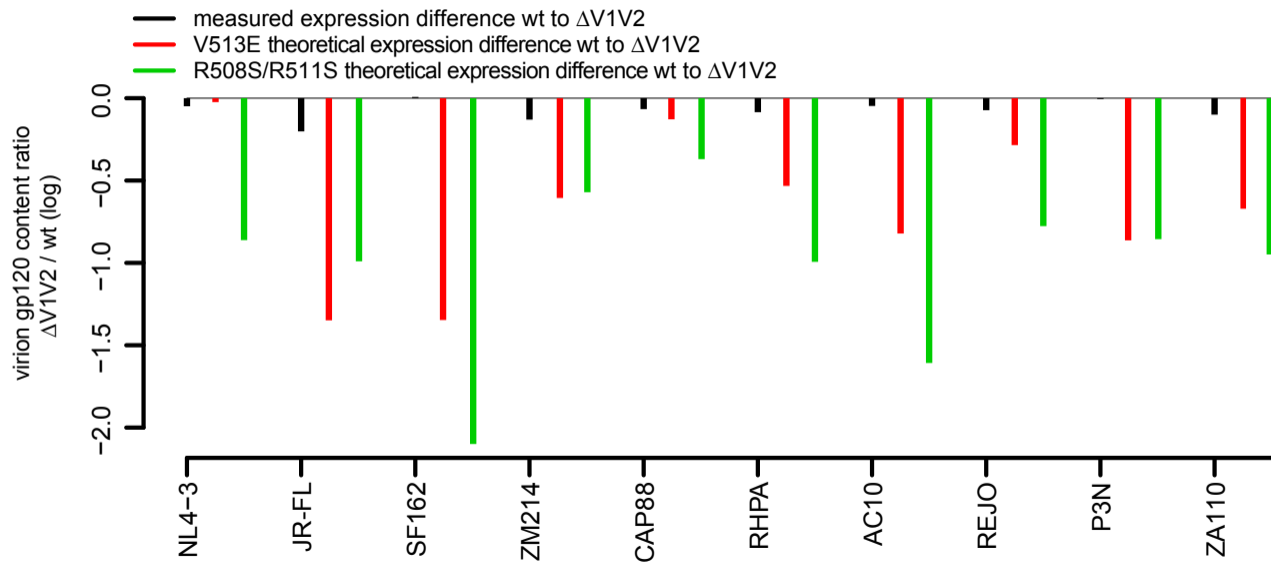

Supplement: S5 Fig — Influence of V1V2-deleted virion gp120 content on estimates of T. Virion gp120 content of V1V2-deleted envs was experimentally determined and compared to wt virion gp120 content. Black bars depict the logarithmic fold difference between V1V2 and wt env expression. Values are means of 2 to 3 independent experiments. The V1V2-deleted envs proved to be expressed and incorporated into virions at similar levels as the wt and were all in the range of 80 to 100% of the corresponding wt envs. The actual V1V2-deleted virion mean trimer contents were then employed in the estimation of T as shown in Fig. 3D. In addition, to control for influences of env expression on our estimates of T, we calculated for each wt – ΔV1V2 env pair the required env content differences that theoretically would be required to cause the observed curve shifts for V1V2-deleted virions in the mixed trimer experiments (Fig. 3B and C), if wt and ΔV1V2 strains would have identical T's. These calculated differences in env expression are shown for the R508S/R511S (green bars) and V513E (red bars) mutations, respectively. While env content certainly influences the estimation of T, we found that only substantial env content differences (between 30 to more than 90% lower than wt for the majority of strains) would result in the observed differences between wt and V1V2-deleted envs. Hence, even for those viruses where expression of the V1V2 deleted env was lower than the wt in our experiments (black bars), this loss proofed not sufficient to induce the shifts in T we observed (Fig. 3D). Thus these analyses confirm that the loss in entry efficiency upon V1V2 deletion we observe is not simply caused by lower envelope content of these virions but indeed indicates a higher T. (PDF) [file ppat.1004595.s005.pdf]

Supplementary Figure S7

A

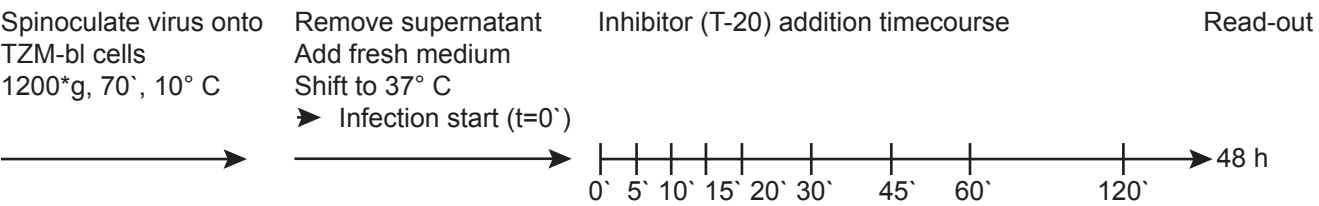

B

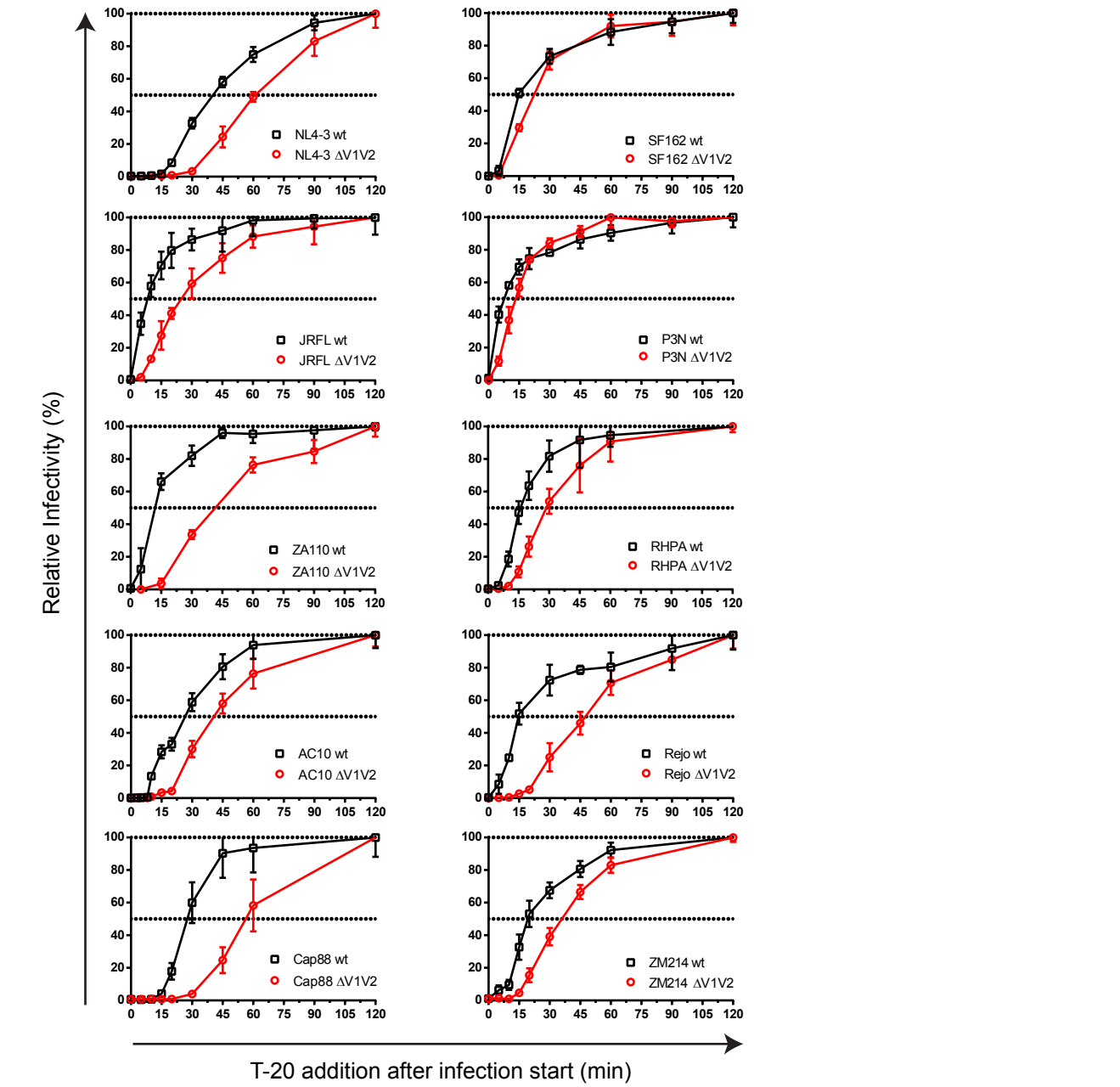

Supplement: S7 Fig — Determination of virus entry kinetics. (A) Scheme of the time-of-inhibitor addition experiment employed to determine virus entry kinetics. In this experiment synchronized infection of TZM-bl cells with pseudoviruses was terminated at consecutive time points by the fusion inhibitor T-20. (B) Virus entry kinetics for the ten pairs of wt and V1V2-deleted strains are depicted as relative infectivity over time of T-20 addition. Infectivity was measured following pseudovirus infection of TZM-bl cells by recording firefly luciferase reporter activity. Infectivity reached after 120 minutes was set as 100% and all data were normalized relative to this value. Data are mean and SD from 2 to 4 independent experiments. From the depicted data, time required for each strain to reach 50% of entry as shown in Fig. 4B of the main text are shown. (PDF) [file ppat.1004595.s007.pdf]

Supplementary Figure S8

A

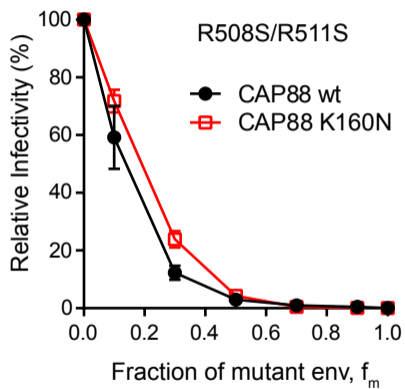

B

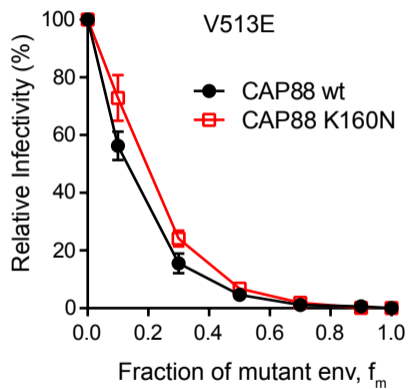

C

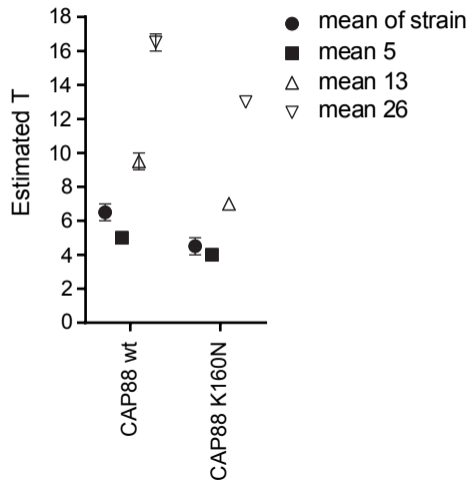

Supplement: S8 Fig — Estimation of T for the CAP88 K160N envelope variant. Relative infectivity of mixed trimer infection experiments of CAP88 wt and the K160N variant using the R508S/R511S (A) and the V513E (B) dominant-negative mutations are shown. Infectivity of pseudotyped virus stocks expressing the indicated ratios of dominant-negative mutant envs was measured on TZM-bl cells. Infectivity of virus stocks containing solely wt envelope were set as 100%. Data depict mean and SD from 2 independent experiments. (C) Estimates of T based on the data depicted in (A) and (B) were derived from 4 different analyses in which we included either the experimentally derived CAP88 mean virion trimer content (identical for both wt and K160N) or assumed trimer contents of 5, 13 and 26 trimers per virion (see also S3A Fig.). Data points depict mean and range of the independent estimates for the R508S/R511S and V513E mutations. (PDF) [file ppat.1004595.s008.pdf]

# Supplementary Figure S9

A

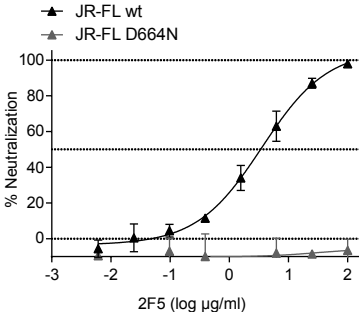

B

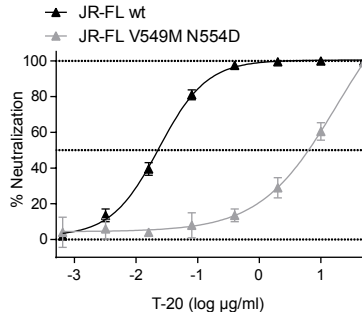

C

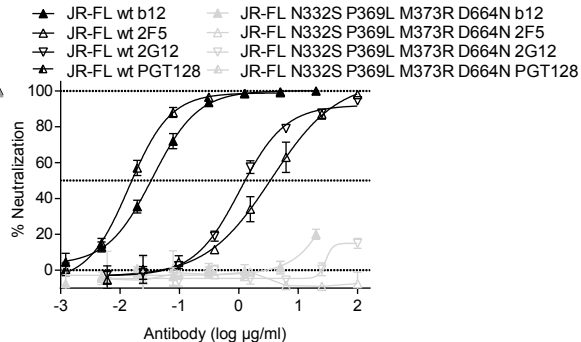

Supplement: S9 Fig — Neutralization escape of JR-FL point mutant envs. (A) to (C): Neutralization of virus by inhibitors was determined by incubating virus stocks with serial dilutions of inhibitors at 37°C for 1h. Subsequently, the virus-inhibitor mixes were transferred to TZM-bl reporter cells and infection was quantified 48h later by luciferase reporter read-out. Infection of cells with mock-treated virus was set to 100% of infectivity (0% neutralization) and % neutralization of virus incubated with different concentrations of inhibitors was calculated in relation to that value. Data depict mean and SD from 2 to 3 independent experiments. (A) Neutralization of JR-FL wt and JR-FL D664N by antibody 2F5. (B) Neutralization of JR-FL wt and JR-FL V549M N554D by T-20. Note that the mutant is still sufficiently sensitive to T-20 to allow assessment of entry kinetics as shown in Fig. 4D and S7A Fig. (C) Neutralization of JR-FL wt and JR-FL N332S P369L M373R D664N by antibodies PGT128, 2G12, b12 and 2F5. (PDF) [file ppat.1004595.s009.pdf]
