# Supplementary material for: Different Infectivity of HIV-1 Strains Is Linked to Number of Envelope Trimers Required for Entry
Source: PLoS Pathog. 2015 Jan 8;11(1):e1004595. doi: 10.1371/journal.ppat.1004595 (PMC4287578; doi:10.1371/journal.ppat.1004595)

Supplementary Figure S2

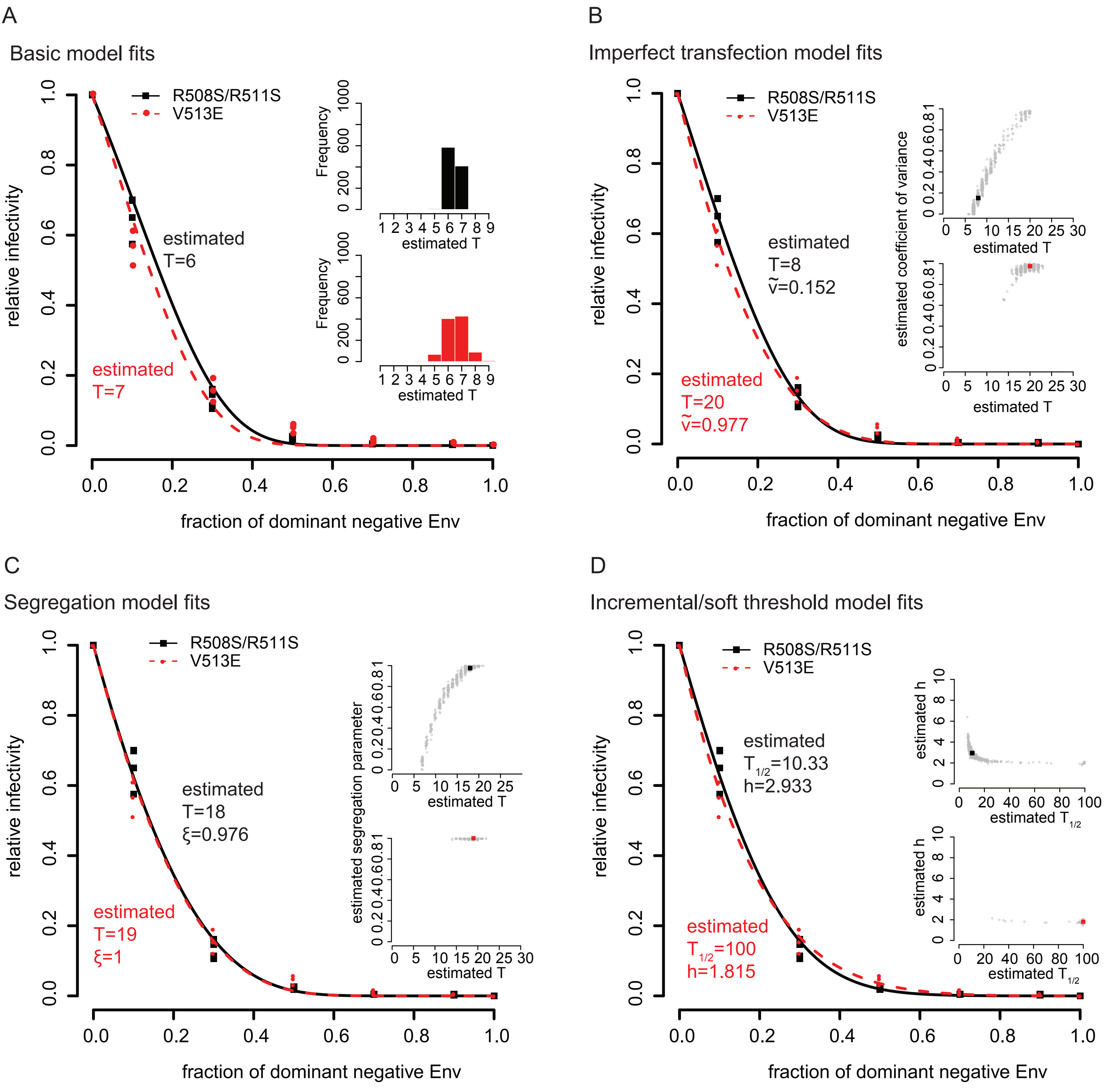

Supplement: S2 Fig — Model extensions for strain CAP88. (A) to (D): To verify if the basic model we used for data analysis is indeed the best choice we performed three extensions of our model and show the results here using strain CAP88 as an example; details of the model fits for all 11 strains are listed in S1 Table. Graphs depict the empirical data of CAP88 shown in Fig. 1B and the according curve fits obtained with our model. The dominant negative mutants are shown in black (R508S/R511S) and red (V513E) respectively. (A) Fit of the basic model, as shown in S1 Fig. (B) Fit of the imperfect transfection model which simulates incomplete transfection of producer cells with env plasmids during production of mixed trimer virus stocks. With this model we estimate both T and the coefficient of variation (). The coefficient of variation ranges from 0 to 1 and is a measure of how different the mixture of envelope proteins inside the transfected cell is in comparison to the env plasmid mixture used to transfect the cell. corresponds to a perfect match (thus this is mathematically equal to the basic model), correspond to cells producing only one type of envelope protein. Note that we obtain very different estimates both for T and the coefficient of variation despite very similar empirical data for the two mutants. The insets show the accuracy of the estimates determined in a bootstrap procedure with 1000 replicates (each gray point represents one bootstrap replicate); the colored dot shows the estimated values of the best fit. (C) Fit of the segregation model, simulating preferential segregation of the wt and mutant envs produced in a transfected cells into homotrimers. From this model we estimate both T and a parameter for the magnitude of the segregation (ξ), ranging from 0 to 1. ξ = 0 corresponds to perfectly randomly mixed trimers (equal to the basic model) and ξ = 1 corresponds to formation of wt and mutant homotrimers only. We obtain very high estimates of T as well as a segregation param [file ppat.1004595.s002.pdf]
