# Supplementary material for: Different Infectivity of HIV-1 Strains Is Linked to Number of Envelope Trimers Required for Entry
Source: PLoS Pathog. 2015 Jan 8;11(1):e1004595. doi: 10.1371/journal.ppat.1004595 (PMC4287578; doi:10.1371/journal.ppat.1004595)

Supplementary Figure S6

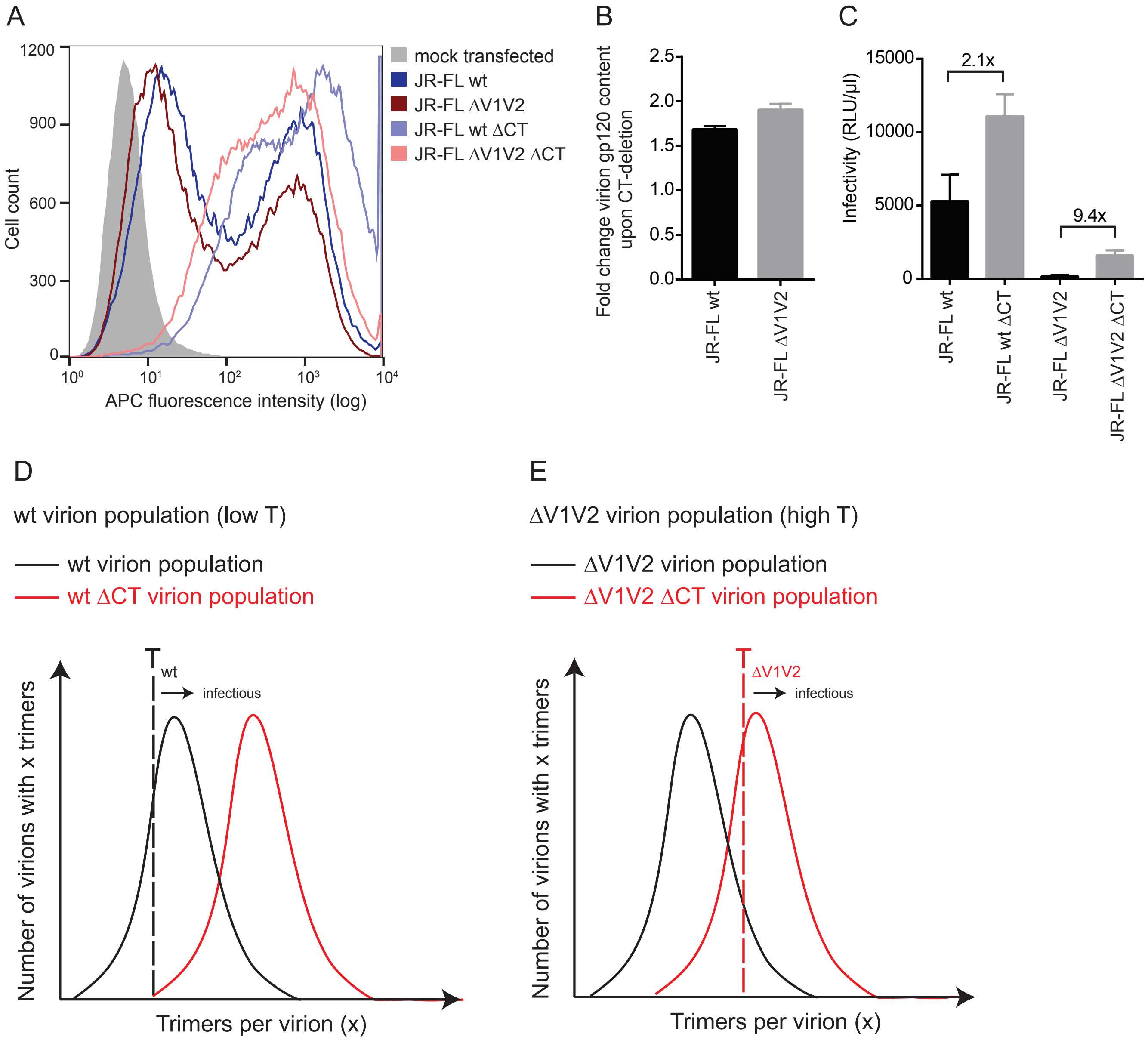

Supplement: S6 Fig — Influence of increased virion trimer numbers on estimates of T and virus population infectivity. (A–C) To further probe the influence of trimer content on T we compared estimates of T and infectivity of viruses on which env content was modulated. According to our model, the amount of functional trimers per virion, in combination with the number of trimers required for entry (T), is a decisive parameter for virion infectivity. (A and B) We produced mutants of JR-FL wt and JR-FL ΔV1V2 with a deletion of the gp41 cytoplasmic tail (CT) by replacing gp41 tyrosine 712 with a STOP codon. (A) 293-T cells were transfected with env encoding plasmids and gp120 expression was determined by cell surface staining with anti-gp120 MAb 2G12 and flow cytometry. Data depict one of two representative experiments. (B) gp120 content measurements for wt and CT deleted JR-FL wt and JR-FL ΔV1V2 were performed by gp120 ELISA. Bars show increase in gp120 content of CT truncated envs compared to full length envelopes. Mean and SD of two independent experiments are shown. As described [39], [40] removal of the endocytosis signal in the CT domain resulted in a higher gp120 content on the surface of JR-FL env-expressing cells and on pseudotyped virions (1.5 and 2-fold higher gp120 content, for JR-FL wt and JR-FL ΔV1V2, respectively. (C) Infectivity of wt and CT deleted virus stocks was determined by titration of virus supernatants on TZM-bl cells and was recorded as activity of the luciferase reporter (relative light unit; RLU) per µl virus stock. Data are mean and SD from 2 independent experiments. The fold change in infectivity upon CT deletion is indicated above the bars for both wt and V1V2 deleted envs. CT-deleted JR-FL wt env infectivity increased by 2.1-fold compared to full-length JR-FL wt, while infectivity of the CT-deleted ΔV1V2 env was elevated 9.4-fold compared to the CT-containing ΔV1V2 env. Thus, the ΔV1V2 env, which has a higher T than the wt env (Fig. 3D), appears to profit more [file ppat.1004595.s006.pdf]
