# Supplementary material for: Different Infectivity of HIV-1 Strains Is Linked to Number of Envelope Trimers Required for Entry
Source: PLoS Pathog. 2015 Jan 8;11(1):e1004595. doi: 10.1371/journal.ppat.1004595 (PMC4287578; doi:10.1371/journal.ppat.1004595)

Supplementary Figure S10

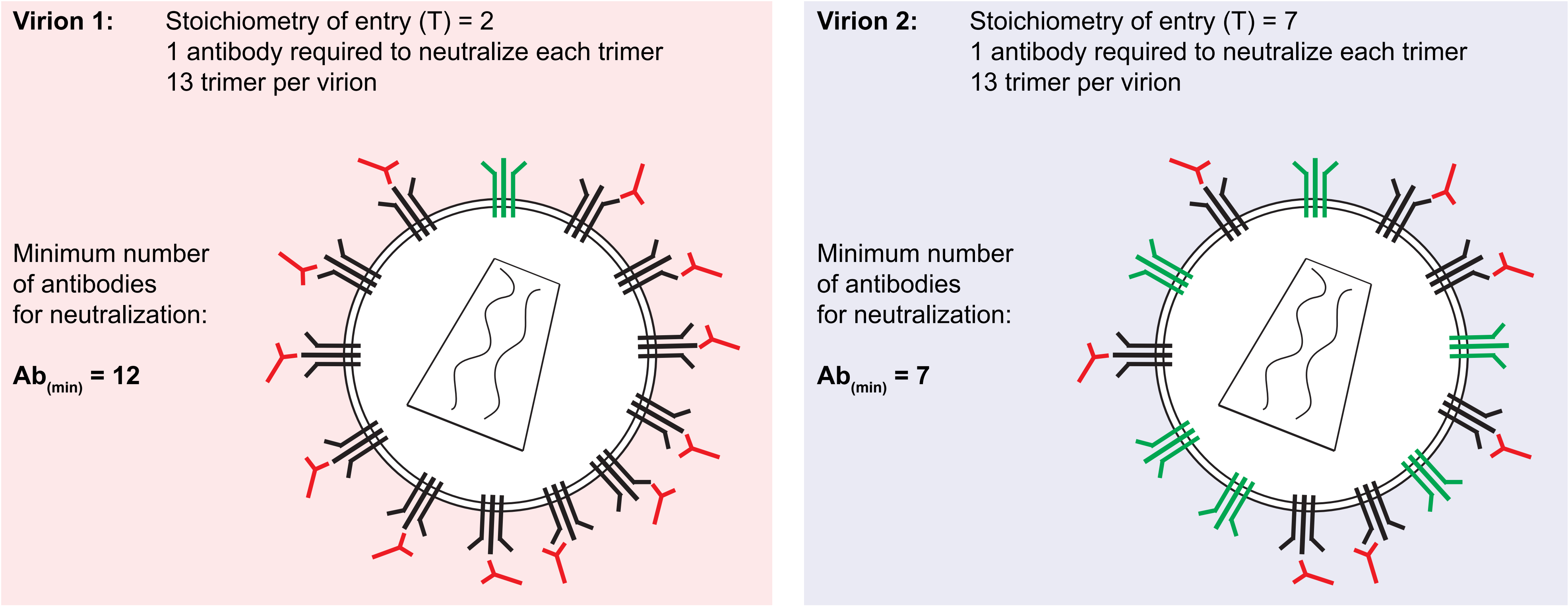

Supplement: S10 Fig — Relation of entry stoichiometry and number of antibodies required for neutralization. Scheme depicting how the stoichiometry of entry influences the efficacy of HIV virion population neutralization by antibodies. Let us assume two virions, one with a stoichiometry of entry of 2, and one with a stoichiometry of entry of 7 (according to Fig. 1C). Both virions carry 13 trimers, and in both cases binding of 1 antibody per trimer is assumed to be sufficient to block trimer functionality. Thus, a minimum of 12 antibodies is required to neutralize the virion with T = 2, while only 7 antibodies are required to neutralize the virion with T = 7, representing a 71% difference in antibody numbers required for neutralization. On a virus population level, these differences in entry stoichiometry may substantially influence the efficacy of neutralizing antibodies [56]. (PDF) [file ppat.1004595.s010.pdf]
