# Supplementary material for: Different Infectivity of HIV-1 Strains Is Linked to Number of Envelope Trimers Required for Entry
Source: PLoS Pathog. 2015 Jan 8;11(1):e1004595. doi: 10.1371/journal.ppat.1004595 (PMC4287578; doi:10.1371/journal.ppat.1004595)
Supplement: S11 Fig — Summary of energies required for membrane fusion and provided by fusion proteins. Values reported in the literature (see main text for details) of the energies required for biological membrane fusion or released by Influenza HA trimers and vesicular SNARE complexes during refolding into the post-fusion conformation are shown. Based on the reported stoichiometries of Influenza virus entry and SNARE-mediated membrane fusion we calculated the total energy contributed by these fusion proteins during the membrane fusion process. In analogy, assuming 40 to 120 KbT for membrane fusion and that 2 to 7 HIV-1 env trimers participate in entry, we derive that each env trimer needs to contribute 6 to 60 KbT during refolding into the post-fusion conformation to mediate membrane fusion. Kb is the Boltzmann constant. (PDF) [file ppat.1004595.s011.pdf]

Supplementary Figure S11

Theoretical considerations of membrane fusion energies

Energy required for membrane fusion:	40 to 120 k_bT
Energy released by Influenza HA trimer refolding:	30 to 60 k_bT
Energy released by SNARE complex refolding:	20 to 65 k_bT

Number of HA trimers estimated to partake in membrane fusion: 3 to 4
> total energy released 90 to 240 k_bT

Number of SNAREs estimated to partake in membrane fusion: 1 to 3
> total energy released 20 to 195 k_bT

Number of env trimers estimated to partake in membrane fusion: 2 to 7

Assuming that the energy required for membrane fusion is 40 to 120 k_bT it follows that each HIV env trimer releases

> 20 to 60 k_bT if T=2
> 6 to 17 k_bT if T=7
