# Supplementary material for: Different Infectivity of HIV-1 Strains Is Linked to Number of Envelope Trimers Required for Entry
Source: PLoS Pathog. 2015 Jan 8;11(1):e1004595. doi: 10.1371/journal.ppat.1004595 (PMC4287578; doi:10.1371/journal.ppat.1004595)
Supplement: S1 Table — Parameters and estimated T values obtained with model extensions. We tested three extensions of our mathematical model (referred to here as “basic model”) to analyze the empirical data shown in Fig. 1B. These extensions include a model accounting for imperfect transfection, a model accounting for envelope protein segregation and a model where we relax the assumption of an absolute threshold for T (soft threshold model). For the first three models we show the estimated T values, as well as the coefficient of variation (imperfect transfection model) and a parameter for segregation (segregation model). For the soft threshold model we show the estimated T1/2, which is the trimer number where 50% infectivity is reached, and the parameter h defining the steepness of the infectivity increase. For all three model extensions we determined the improvement of the fit compared to the basic model with an F-test and show the corresponding p-value as a measure of significance (p<0.05 means significant improvement; see also S2 Fig. for details of the alternative model fits.) These significantly better fits are not necessarily an indication of biologically more meaningful results. Suitability of model predictions need to be decided on by taking the question being addressed into account and whether the estimated parameters are in accordance with the biological system investigated. In our case, all models except the basic model yielded in several cases T estimates that are far higher than what can be expected in the context of HIV infection. In addition, the basic model was the only one that yielded comparable T estimates for both dominant negative mutants in the majority of the probed strains. (PDF) [file ppat.1004595.s012.pdf]

Supplementary Table S1

Strain	Mutation	Basic model	Imperfect transfection model			Segregation model			Soft threshold model		
		estimated T	estimated T	estimated coefficient of variation	p value of comparison to basic model (F-test)	estimated T	estimated segregation parameter	p value of comparison to basic model (F-test)	estimated T_1/2	estimated h	p value of comparison to basic model (F-test)
NL4-3	R508SR511S	4	12	0.952	<0.0005	12	0.984	<0.0005	7.986	1.328	<0.0005
NL4-3	V513E	7	18	0.977	<0.0005	18	1	<0.0005	20.108	1.596	<0.0005
JR-FL	R508SR511S	2	8	0.98	<0.0005	8	1	<0.0005	2.134	1.232	<0.0005
JR-FL	V513E	2	8	0.953	<0.0005	8	1	<0.0005	79.074	0.463	<0.0005
SF162	R508SR511S	5	12	0.859	<0.0005	13	0.993	<0.0005	5.834	1.643	<0.0005
SF162	V513E	4	12	1	<0.0005	11	1	<0.0005	100	0.576	<0.0005
ZM214	R508SR511S	2	3	0.084	<0.0005	3	0.235	0.094	1.956	7.578	0.042
ZM214	V513E	2	7	0.951	<0.0005	7	1	<0.0005	100	0.525	<0.0005
CAP88	R508SR511S	6	8	0.152	<0.0005	18	0.976	0.001	10.329	2.933	<0.0005
CAP88	V513E	7	20	0.977	<0.0005	19	1	<0.0005	100	1.815	<0.0005
RHPA	R508SR511S	2	8	0.93	<0.0005	8	0.972	<0.0005	4.17	0.718	<0.0005
RHPA	V513E	3	10	1	<0.0005	9	0.99	<0.0005	12.209	0.668	<0.0005
AC10	R508SR511S	3	7	0.568	<0.0005	5	0.509	0.412	2.454	2.94	0.038
AC10	V513E	2	7	0.979	<0.0005	7	1	<0.0005	3.223	0.329	<0.0005
REJO	R508SR511S	1	2	0.262	<0.0005	2	0.464	0.055	94.882	0.061	0.044
REJO	V513E	2	6	0.872	<0.0005	6	0.966	<0.0005	3.541	0.519	<0.0005
P3N	R508SR511S	2	10	0.986	<0.0005	10	1	<0.0005	1.867	1.283	<0.0005
P3N	V513E	2	10	0.998	<0.0005	10	1	<0.0005	1.954	0.825	<0.0005
ZA110	R508SR511S	2	4	0.378	<0.0005	4	0.598	0.081	1.364	2.932	<0.0005
ZA110	V513E	2	8	1	<0.0005	7	0.969	<0.0005	1.357	0.872	<0.0005
BG505	R508SR511S	3	4	0.165	<0.0005	5	0.567	0.111	2.593	2.89	0.001
BG505	V513E	2	7	0.933	<0.0005	7	1	<0.0005	1.878	1.132	<0.0005
